# In-depth analysis on PTB7 based semi-transparent solar cell employing MoO_3_/Ag/WO_3_ contact for advanced optical performance and light utilization

**DOI:** 10.1038/s41598-023-34507-y

**Published:** 2023-05-09

**Authors:** Erman Çokduygulular, Çağlar Çetinkaya, Serkan Emik, Barış Kınacı

**Affiliations:** 1 Department of Engineering Sciences, Faculty of Engineering, Istanbul University-Cerrahpaşa, 34320 Istanbul, Turkey; 2 Physics Department, Faculty of Science, Istanbul University, 34134 Istanbul, Turkey; 3 Department of Chemical Engineering, Faculty of Engineering, Istanbul University-Cerrahpaşa, 34320 Istanbul, Turkey; 4 Department of Photonics, Faculty of Applied Sciences, Gazi University, 06500 Ankara, Turkey; 5 Photonics Application and Research Center, Gazi University, 06500 Ankara, Turkey

**Keywords:** Solar cells, Micro-optics

## Abstract

Novel semi-transparent organic solar cells (ST-OSC) can be designed with high average visible transmittance (AVT) while at the same time exhibiting superior photovoltaic performance. This reach requires their design to be based not only on conventional window applications but also on functional industrial applications that require exceptional optical performance. In ST-OSC, high AVT can be achieved by photonic-based dielectric/metal/dielectric (DMD) transparent contact engineering. Functional optical modification can also be made with a fine-tuned design of DMD that includes a light management engineering-based approach. Thus, ST-OSCs can be suitable for aesthetic, colourful and decorative industrial windows that provide natural lighting. In this study, we determined optimal ST-OSCs based on a novel PTB7:PC_71_BM polymer blend with MoO_3_/Ag/WO_3_ asymmetric DMD top contact by examining extraordinary optical properties such as AVT, colour rendering index, correlated colour temperature and colour perception over 10 thousand designs. In addition to determining the optimality and extraordinary optical limits for PTB7, we also evaluated the photon-harvesting and photovoltaic performance of ST-OSCs from external quantum efficiency and quantum utilization efficiency. In optimal situations, ST-OSCs offering 48.75% AVT, 99.08 CRI, and sky-blue colours were designed and determined to generate short-circuit current densities of 9.88 mA·cm^−2^, 13.64 mA·cm^−2^, and 13.06 mA·cm^−2^, respectively.

## Introduction

Organic solar cells (OSCs) are very promising due to the advantages and potential offered by their properties such as processability, lightness, mechanical flexibility, cost-effective fabrication and tunable optical transparency compared to conventional inorganic cells^1^. In recent years, the focus has been on developing innovative donor and acceptor molecules that will provide low energy loss in the active layer to achieve high efficiency. In particular, 19% power conversion efficiency (PCE) has been achieved using non-fullerene acceptors (NFA) with easily adapted chemical structures and finely tuned band gaps, energy levels and crystallization^1^. The structural arrangement of NFA is critical for the light absorption, phase separation and charge transport properties of photovoltaic organic mixtures with electron donors, which determines the OSC’s PCE. Li et al. have considered an approach to creating a refined fibril structure of a recently reported high-performance NFA L8-BO with the aid of the fused-ring solvent additive 1-fluoronaphthalene (FN). In this way, they improved from 16.0 to 19% PCE by improving light absorption, charge transport and collecting properties^2^. Gao et al., on the other hand, increased the efficiency above 19% in planar mixed heterojunction (PMHJ) OSC by making asymmetric selenium substitution in the construction of BS3TSe-4F, which is a pseudosymmetric electron acceptor^1^. In addition to great PCE improvement, an environmentally friendly production can be achieved with the use of so-called green solvents such as ortho-xylene (o-XY) during the fabrication processes of OSCs processed with halogenated solvents such as chlorobenzene, which is considered very dangerous for human health and the environment based on environmental impact^3^.

OSCs can exhibit semi-transparent (ST) optics. In particular, the fact that the organic materials forming the active region of OSCs have a higher absorption coefficient allows them to be designed more efficiently and useful^4^. Therefore, OSCs have the potential to be fabricated as efficient, ultra-thin, and hence ST^4–6^. Due to the diversity in organic materials, ST-OSCs can be produced in various colours^4,5,7^. ST-OSCs can be suitable for aesthetic decorative products and can be used in various building architectures and industrial applications that can be integrated into windows. These applications can provide natural illumination to the indoor environment while offering multi-colouration, especially for automobiles and buildings integrated with photovoltaics (AIPV and BIPV, respectively).

The design of ST-OSCs aims to absorb electromagnetic radiation outside the visible region optimally and to be as transparent as possible outside this range^8,9^. For this purpose, new donor and acceptor molecules with narrow band gaps have been developed to enhance the absorption of near-infrared (NIR) regions and ST-OSCs have been optimized by designing new transparent conductive films or optical coupling layers to modulate light transmission^5–7,10,11^. Recent years have seen tremendous progress in ST-OSCs: PCEs have been improved from 5% to over 13%, while high AVTs of over 25% have been maintained^12,13^.

Integrating different photonic-based systems into the solar cell (SC) structure without modifying the structure of the organic material forming the active region in ST-OSCs also enables the achievement of ST requirements^6^. For ST, dielectric/metal/dielectric (DMD) transparent contact designs with high optical transmittance and electrical conductivity, low turbidity, excellent flexibility, easy fabrication and great compatibility with different substrates can be used^6,7,14–16^. In the DMD design, a thin metal material is sandwiched between two anti-reflective dielectrics, and the thickness of these layers can modify the optical properties. The DMD can be designed with low reflectivity of the transparent electrodes, and thus, the photon absorption of the device can be carefully tuned to allow sufficient light to pass through the device^16,17^.

Since there is a balance between light absorption and transmission in ST optoelectronic devices, one of the vital problems in optimising ST-OSCs is to design with this optical optimization in mind. Furthermore, for optical modification in ST-OSC, changing the optical characteristics of the organic material forming the active region also affects the electrical performance. This can adversely affect photovoltaic performance. For that reason, it is quite a functional approach to modify the propagation of the electromagnetic wave in the ST device without modifying the active organic material^6,7,14–16^. For this purpose, approaches based on light management engineering have been used to optimize the optical properties of ST-OSC^18–20^. Among these approaches, it is rather advantageous to use Transfer Matrix Method (TMM) for enhanced and modifiable optical properties^4,6,7,14,21^.

Within the scope of the study, we determined the optimal structure parameters by integrating an asymmetric MoO_3_/Ag/WO_3_ DMD transparent contact system into OSCs whose active region is formed by a novel polymer PTB7:PC_71_BM bulk-heterojunction (BHJ) blend with high average visible transmittance (AVT) and optical properties such as colour rendering index (CRI), correlated colour temperature (CCT) and colour perception. In this strategy, a light management engineering-based approach is pursued with TMM calculations. The optimal structures’ external quantum efficiency (EQE) and quantum utilization efficiency (QUE) characteristics, photovoltaic performances and light harvesting properties are examined in detail based on the extraordinary optical properties.

## Result and discussion

**Figure 1:**
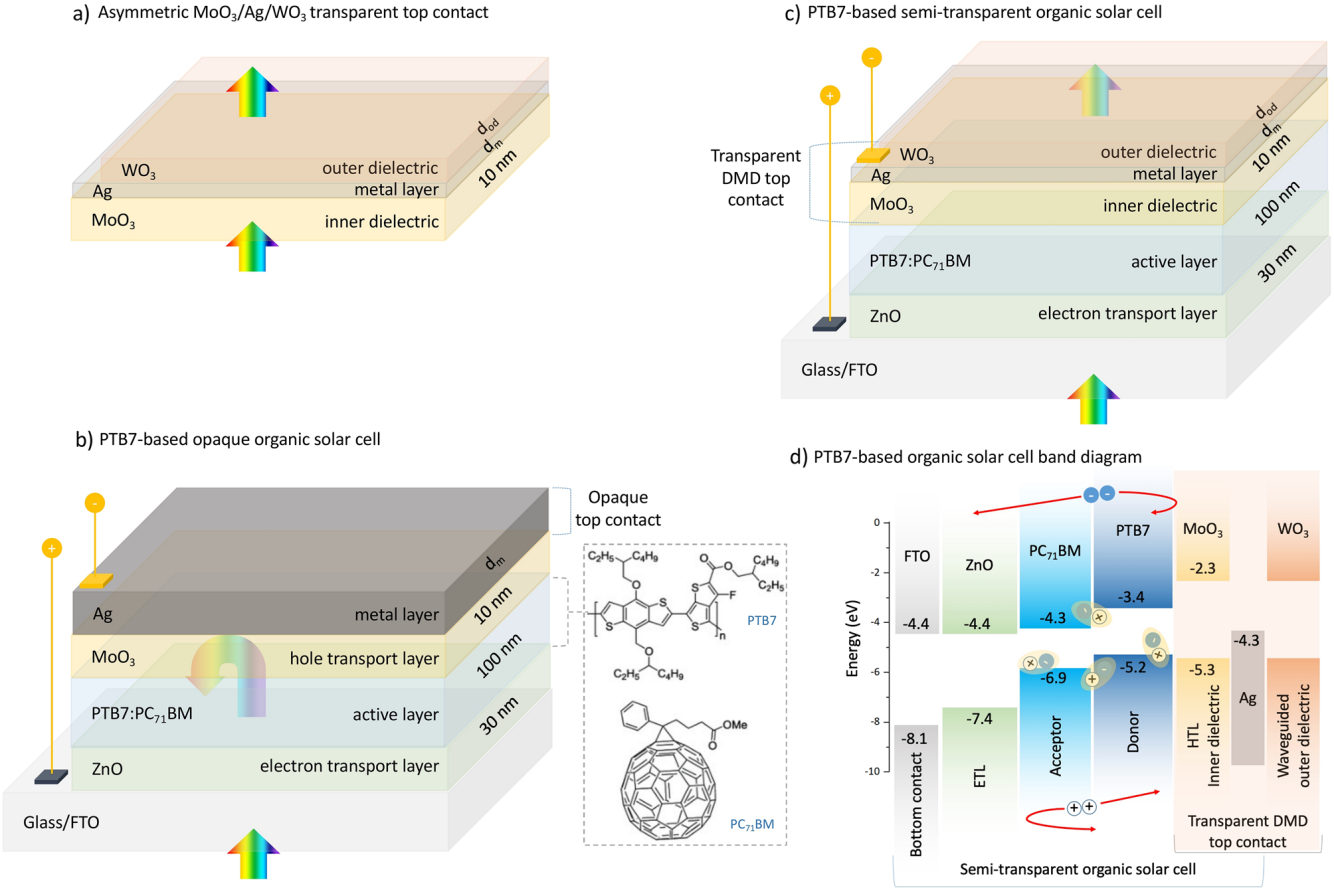
PTB7-based organic solar cell structures. (**a**) Asymmetric MoO_3_/Ag/WO_3_ transparent contact system, (**b**) structure of opaque PTB7-based organic solar cell with thick Ag top contact and chemical structures of PTB7 and PC_71_BM, (**c**) PTB7-based semi-transparent organic solar cell with asymmetric MoO_3_/Ag/WO_3_ transparent contact system, (**d**) band arrangement of organic solar cell.

The OSCs examined in this study were designed with inverted structure architecture and polymer BHJ active region. The detailed structure and band arrangement of OSCs are presented in Fig. 1 and the material and method section. DMD transparent contact systems should be designed to be fine-tuned based on the application potential of the ST-OSCs into which they are integrated. For ST-OSCs to be used in window applications, optical properties of the DMD system should be at high AVT and colour coordinates close to the Planckian locus or D65^6,7,14,22^. Neutral colour, high CRI and CCT values are other design parameters that should be targeted for ST-OSCs^7,14^. These outstanding optical properties are related to the wavelength dependence of the refractive indices and extinction coefficients of all layers forming the ST-OSC. They are also directly related to the thickness of the upper and lower dielectric materials and the ultrathin metallic layer in the integrated DMD system. Therefore, the AVT, CRI, colour and CCT characteristics of the integrated asymmetric MoO_3_/Ag/WO_3_ (MAW) (10/d_m_/d_od_ nm) transparent top contact system have been evaluated for PTB7-based OSC for ST requirement.

### Average visible transmittance of PTB7-based ST-OSC with MoO_3_/Ag/WO_3_

The conversion from opaque to ST optical properties in PTB7-based OSC was realized by integrating an asymmetric MAW transparent contact system. In the PTB7-based ST-OSCs with asymmetric MAW transparent contact system investigated in this study, the MoO_3_ hole transport layer (HTL) acting as the inner dielectric not only provides a hole injection into the organic layers but also acts as an anti-reflection layer for reflective metals such as Ag and Au with a high refractive index^14,23^. By using WO_3_ as the outer dielectric, the radiation loss from the surface plasmon in the Ag layer is suppressed; thus, the DMD structure’s transmittance can be modified by the thickness of WO_3_^23^.

**Figure 2:**
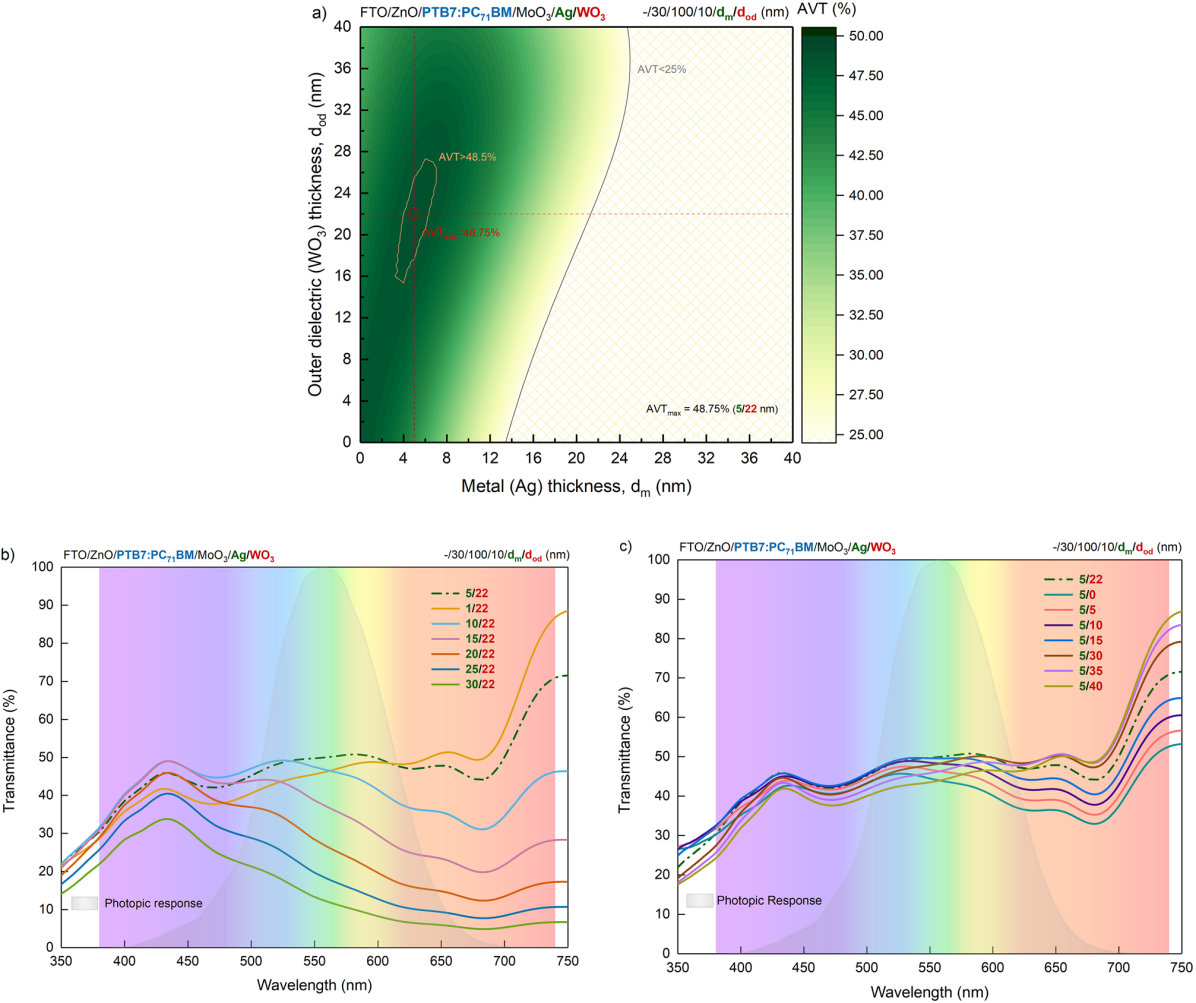
Transparency properties of PTB7-based semi-transparent organic solar cell with asymmetric MoO_3_/Ag/WO_3_ transparent contact system. (**a**) A mapping of the calculated AVT values for PTB7-based OSC over d_m_ and d_od_ of integrated asymmetric MoO_3_/Ag/WO_3_ (10/d_m_/d_od_ nm) DMD transparent contact system, optical spectra calculated according to the change of (**b**) d_m_ and (**c**) d_od_. d_m_ in the 4–8 nm and d_od_ in the range of 16–27 nm range are greater than 48.5% (represented by the area surrounded by yellow in (**a**)). The detailed representation is in Supplementary Figs. 2–4 for absorbance, reflectance and transmittance, respectively.

The reflective behaviour of the metals used in the DMD system in VR and the variation of their electrical properties depending on their thickness create a delicate balance between conductivity and transmission of the electromagnetic wave. This is the most critical design parameter for ST-OSCs having DMD transparent contacts. In particular, the material forming the metal layer and its thickness require significant optimization. The requirement for transparent electrodes to have high transmittance and low reflectance must be carefully optimized to allow sufficient light to pass through for photon absorption and reflection. Thus, this study presents an AVT evaluation of the asymmetric MAW (10/d_m_/d_od_ nm) transparent contact system integrated into the PTB7-based OSC based on d_m_ and d_od_ values. The investigation is based on a light management-based approximation for high transmittance and hence high AVT requirement, and the optical spectra, such as reflectance, transmittance and absorption of the PTB7-based OSC with the TMM, are calculated. Figure 2a–c shows mapping of the calculated AVT for the PTB7-based OSC based on the d_m_ and d_od_ values of the integrated asymmetric MAW transparent contact system and the calculated optical spectra based on the variation of d_m_ and d_od_, respectively.

AVT of PTB7-based ST-OSC with d_m_ variation in the 1–12 nm range and d_od_ variation in the 0–40 nm range is greater than 25%, which is the lower limit for window applications. This shows that the asymmetric MAW system is a highly suitable transparent contact for PTB7-based ST-OSC. The maximum AVT value of 48.75% is obtained at d_m_ = 5 nm and d_od_ = 22 nm and is represented by the intersection of the horizontal and vertical dashed lines in Fig. 2a. With such optimal thickness values, it offers a considerably higher AVT compared to PTB7-based ST-OSCs of different structures with high AVTs obtained in the literature^5,24^.d_m_ has a much more significant effect on AVT values than d_od_. Especially for d_m_ larger than 15 nm, AVT shows a drastic decreasing trend and PTB7-based OSC shows opaque characteristics after 60 nm. The variation of the spectra over thickness is presented for the optimal AVT by the curves represented by the dashed lines in Fig. 2a. In particular, with d_m_, the transmittance in the NIR decreases significantly in the VR, while with d_od_, a change is observed in the long wavelength region of the VR and the NIR. Therefore, the transmittance values of the WO_3_ dielectric layer in the middle of the VR and in the short wavelength region are not substantially affected. The AVT is significantly enhanced by increasing the d_od_ up to 22 nm for a constant d_m_. This is because the transparency, reduced by the strong electric field and the plasmonic effect observed in metals caused by surface charges, is improved by a thicker outer WO_3_ dielectric layer^14,23^. As such, the outer dielectric layer WO_3_ also acts as a waveguide in the ST-OSC.

### Colour rendering index of PTB7-based ST-OSC with MoO_3_/Ag/WO_3_

**Figure 3:**
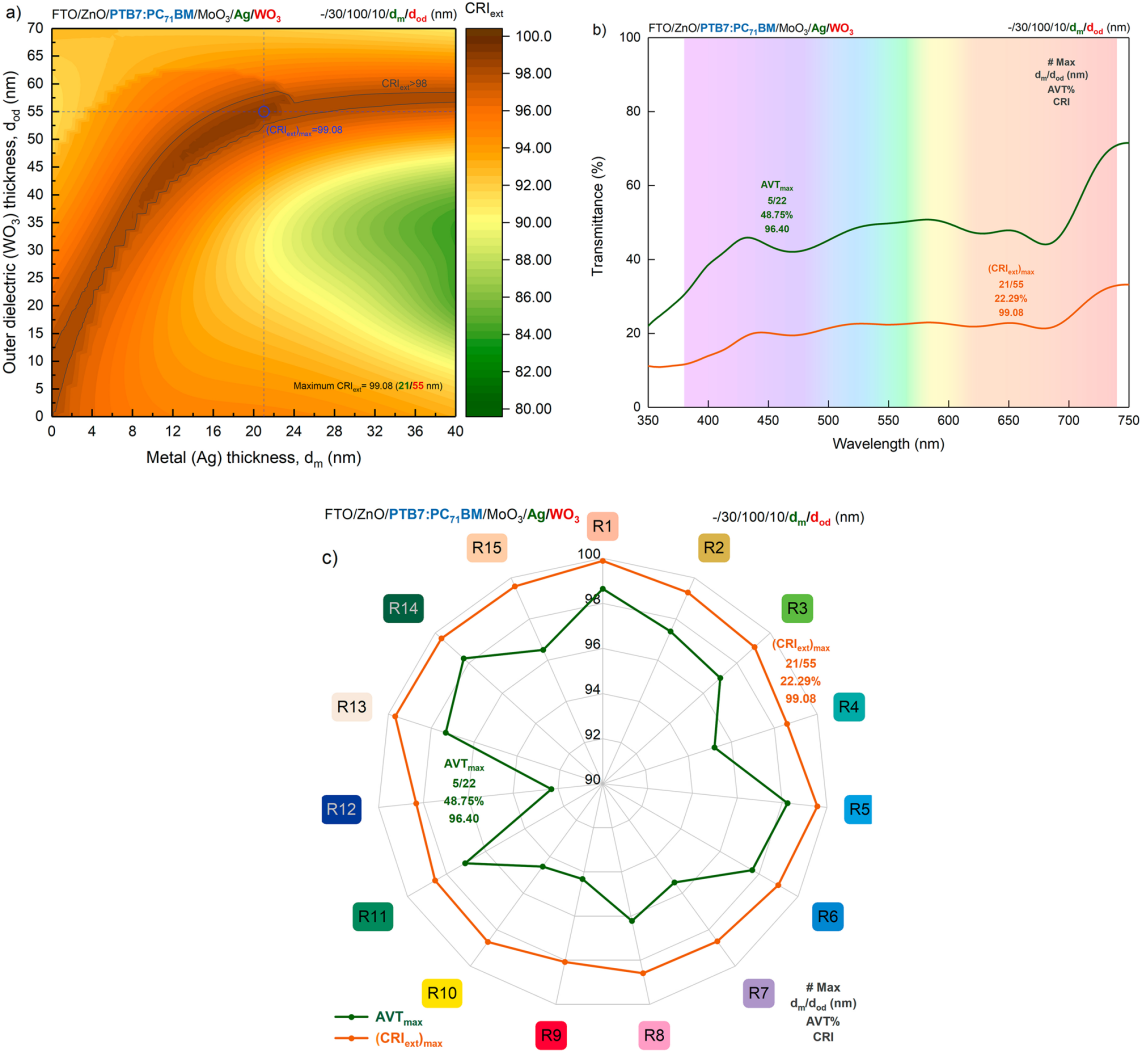
Colour rendering properties of PTB7-based semi-transparent organic solar cell with asymmetric MoO_3_/Ag/WO_3_ transparent contact system. (**a**) CRI_ext_ distribution calculated for PTB7-based OSC over d_m_ and d_od_ values of integrated asymmetric MoO_3_/Ag/WO_3_ (10/d_m_/d_od_ nm) DMD transparent contact system and (**b**) optical spectra of optimal structures with maximum average visible transmittance and maximum colour rendering index. (**c**) Test colour sample values of optimal PTB7-based ST-OSCs offering maximum AVT and CRI_ext_. The presented test colour samples are represented in their true colours and are given by R. The detailed representation is in Supplementary Fig. 5.

Evaluating the CRI values for an ST optoelectronic device is essential for window applications and indoor lighting. The ability of a light source to accurately regenerate the colours of the illuminated object is determined by the CRI. The CRI_ext_ distribution of the PTB7-based ST-OSC, in which the asymmetric MAW (10/d_m_/d_od_ nm) transparent contact system is integrated, calculated by TMM based on d_m_ and d_od_ values, the transmittance spectra and test colour samples (TCS) values of the optimal structures determined based on CRI_ext_ by AVT are presented in Fig. 3a–c, respectively.

CRI_ext_ of PTB7-based ST-OSC is 80 and above with the change of d_m_ in the range of 0–40 nm and d_od_ in the range of 0–70 nm. Besides, CRI_ext_ of 90 and above can be obtained for a wide range of d_m_ and d_od_. This shows that the PTB7-based ST-OSC with asymmetric MAW transparent contact system has superior performance for in-door lighting and window applications. Because CRI values of 80 and above are acceptable for optoelectronic devices for in-door and industrial applications, it is especially crucial to acquire CRI values of 90 and above for in-door exhibitions and showcase lighting where colour appearance is important^7,14,25^. The maximum CRI_ext_ value is 99.08, obtained at d_m_ = 21 nm and d_od_ = 55 nm. Therefore, an ST device with remarkable optical performance can be designed by integrating PTB7 and MAW transparent contact systems.

The AVT value of the PTB7-based ST-OSC with asymmetric MAW (10/21/55 nm) transparent contact system with maximum CRI_ext_ value is 22.29%. The AVT sacrifice for the optimal case can be seen in Fig. 3b. Since CRI_ext_ is an average evaluation over TCS, it is determined by weighting the specific spectra of each TCS. Thus, structures that offer a flat transmittance spectrum in VR have a high CRI_ext_. The transmittance spectrum of the PTB7-based ST-OSC with maximum CRI_ext_ does not vary substantially over the entire VR and the transmittance value is almost the same for all wavelengths. In contrast, the transmittance spectrum of the PTB7-based ST-OSC with maximum AVT shows a significant decrease around 450 nm and 750 nm, and though the AVT is high, the CRI_ext_ value is relatively lower. For the ST-OSC with maximum AVT, the CRI_ext_ is 96.40, still providing a reasonably strong optical performance.

### Colour perception of PTB7-based ST-OSC with MoO_3_/Ag/WO_3_

**Figure 4:**
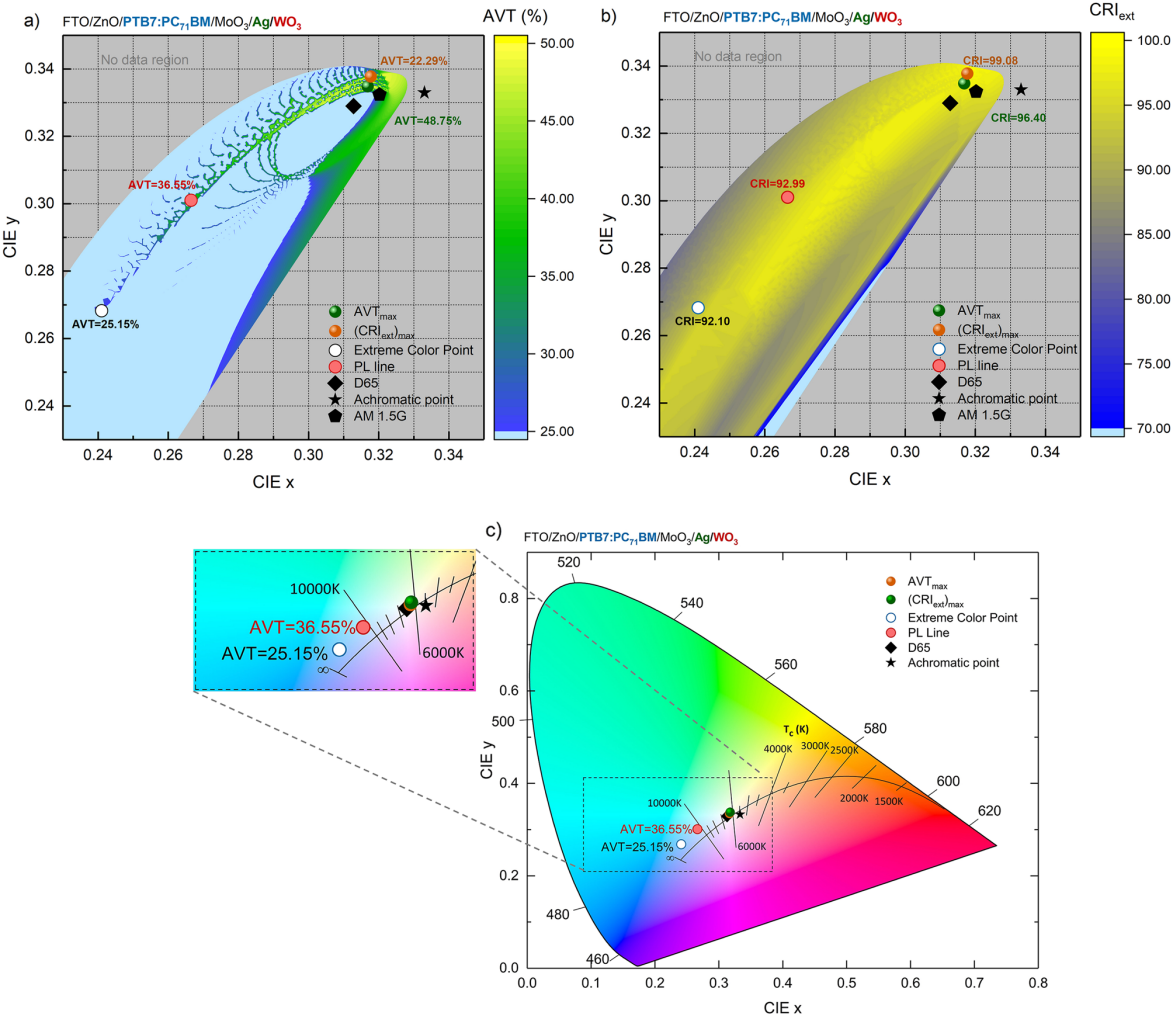
Colour perception properties of PTB7-based semi-transparent organic solar cell with asymmetric MoO_3_/Ag/WO_3_ transparent contact system. (**a**) AVT, (**b**) CRI_ext_ distributions according to CIE x and y coordinates of PTB7-based ST-OSCs with asymmetric MoO_3_/Ag/WO_3_ (10/d_m_/d_od_ nm) DMD transparent contact system and, (**c**) CIE x and y colour coordinates of optimal structures. d_m_/d_od_ = 15/22 nm where the colour coordinates change along the PL line, the CIE x and y are 0.2665 and 0.3011, respectively.

Although AVT, which is examined for ST target, and CRI_ext_, investigated for colour efficiency, are essential for lighting in indoor applications, colour properties are important for exterior architecture and indoor lighting. Because effective architectural and industrial designs should be made for BIPV and AIPV applications with ST-OSCs. The asymmetric MAW transparent contact system is integrated with PTB7-based OSC and colour coordinates are calculated over d_m_ and d_od_ with TMM in this research. The mapping of the CIE x and y coordinates of the designed ST-OSCs with AVT and CRI_ext_ distributions and the change of CIE x and y colour coordinates are given in Fig. 4a–c, respectively.

Figure 4a shows the CIE x and y colour coordinates and the values of AVT that can be obtained for a PTB7-based ST-OSC with an asymmetric MAW transparent contact system. The light grey area in the graph is the region where AVT less than 25% can be achieved. It is not possible to design a PTB7-based ST-OSC in the colour region represented by the grey area. Thus, the colour change of a PTB7-based ST-OSC with the DMD can only be performed from the neutral colour region to the blue region. The colour coordinates of the optimal structures presented for AVT and CRI_ext_ are close to each other and to the AM 1.5G colour coordinates. Therefore, a ‘colourless’ PTB7-based ST-OSC with neutral colour perception can be obtained for industrial designs requiring both high AVT and high CRI_ext_. The CCTs of the optimally presented structures are 5100 K and 5055 K, respectively, and their colour-related optical parameters are given in Table 1.

**Table 1:** Optical properties of optimally designed PTB7-based semi-transparent organic solar cells with asymmetric MoO_3_/Ag/WO_3_ transparent contact system.

Characteristic	PTB7-based ST-OSC with MoO_3_/Ag/WO_3_ (10/d_m_/d_od_ nm) transparent top contact optical parameters							
	MoO_3_/Ag/WO_3_ (10/d_m_/d_od_ nm)	AVT (%)	CRI_ext_	CIE x	CIE y	CCT (K)		∆_u,v_
AVT_max_	5/22	**48.75**	96.40	0.3168	0.3349	5100	Daylight	0.00456
(CRI_ext_)_max_	21/55	22.29	**99.08**	0.3177	0.3378	5055	Daylight	0.00037
PL line	15/22	36.55	92.99	**0.2665**	**0.3011**	8280	Overcast	0.00635
Extreme colour	20/19	25.15	92.10	**0.2410**	**0.2683**	**12,792**	**Sky blue**	0.00754

Significant values are in bold.

The colour coordinates that can only be shifted to the blue region for colour modification in PTB7-based ST-OSC can be changed along the Planckian locus (PL) up to the 25% AVT limit. For the most colour-modifiable structure (d_m_/d_od_ = 20/19 nm) at the 25% AVT limit, the colour coordinates that we refer to as ‘extreme colour’ (CIE x and y are 0.2410 and 0.2683, respectively) (other optical properties are presented in Table 1). Furthermore, with the asymmetric MAW transparent contact system for PTB7-based ST-OSC, CCTs can be modified from 5000 K daylight region to 12,792 K region in sky blue and beyond. The colour coordinates of the optimally and colourfully designed PTB7-based ST-OSCs are given in Fig. 4c in the CIE1931 chromaticity diagram. As d_m_ increases, the value of the colour coordinates decreases and shifts towards the blue region due to the reduction in transmittance and enhancement of reflection in the long wavelength range of the ST-OSC, particularly in the VR regime. This effect has been previously observed by us and is a methodology used to change the colour of an ST-OSC, which is coloured by the nature of the polymer, to exhibit a neutral colour^6^.

Figure 4b shows how the distribution for CRI_ext_ can be obtained with CIE x and y colour coordinates in a PTB7-based ST-OSC with an asymmetric MAW transparent contact system. It can be seen that a CRI_ext_ of 80 and above can be obtained for the colour region where ST properties can be achieved. Therefore, the DMD system does not deteriorate the colour rendering of the PTB7-based ST-OSC. The ST-OSC designed with donor PTB7 and acceptor PC_71_BM polymers with an asymmetric MAW transparent contact system is understood to offer outstanding optical properties. However, the optimal structures presented in the study have determined what type of transparent contact system should be designed with the required optical properties.

### Photovoltaic performance of PTB7-based ST-OSC with MoO_3_/Ag/WO_3_

**Figure 5:**
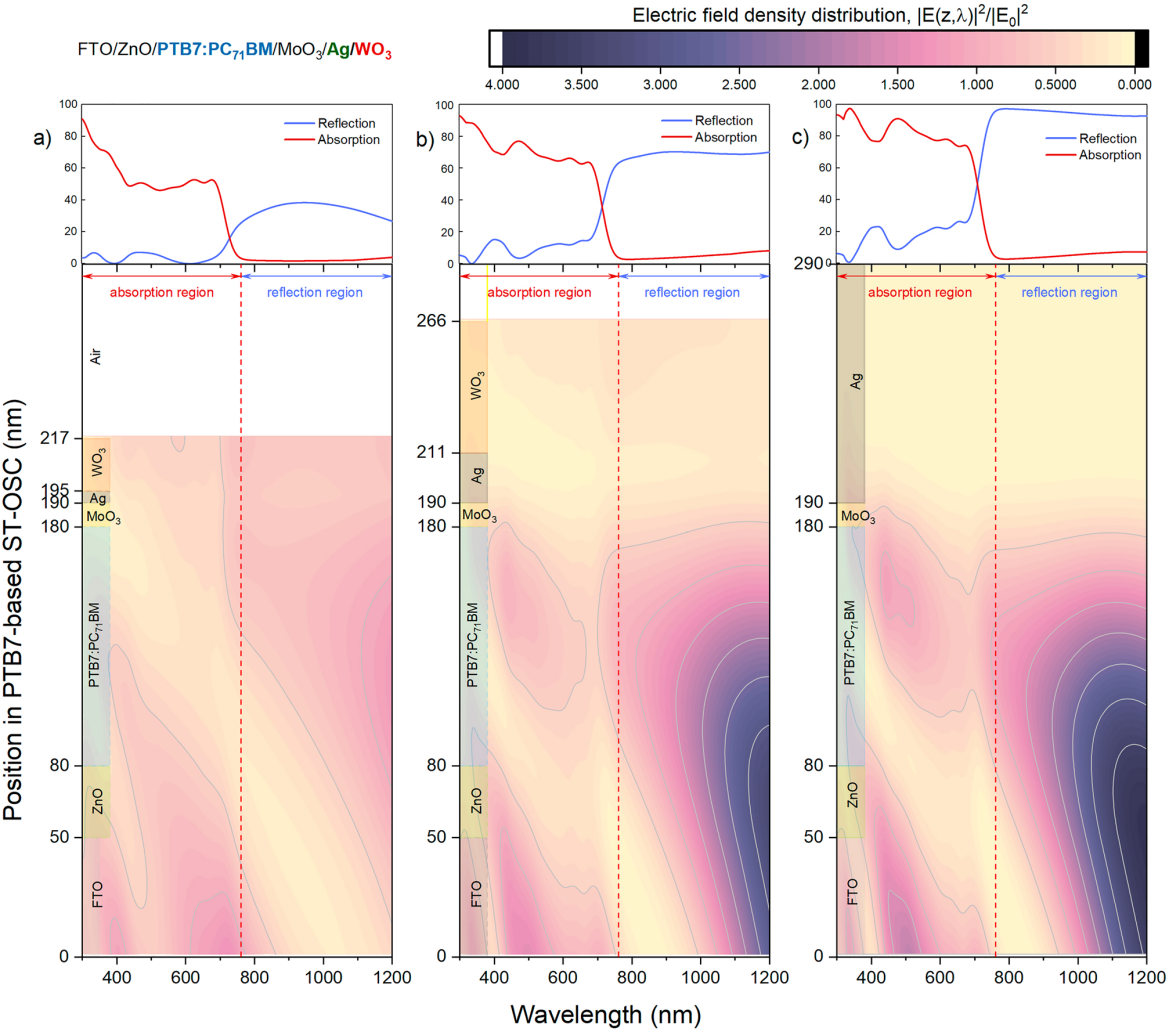
Electric field intensity distribution in PTB7-based organic solar cells with asymmetric MoO_3_/Ag/WO_3_ transparent top contact system and opaque Ag top contact. The distribution of electric field intensities in the structure according to the wavelength of PTB7-based OSCs with asymmetric MoO_3_/Ag/WO_3_ transparent contact and (**c**) opaque Ag top contact, which is determined optimally based on (**a**) AVT and (**b**) CRI_ext_, are presented. In addition, reflection and absorption characteristics are presented on the upper part for each designed OSC.

Modifying the optical properties of PTB7-based ST-OSC with an asymmetric MAW transparent contact system also affects the electric field distribution in the SC. Thus, the mechanisms affecting photon harvesting in ST-OSCs with different optical properties will exhibit differences, and the photovoltaic performance characteristics will change. In the present study, the position and wavelength-dependent electric field distributions of the OSCs analysed by light management engineering approaches were determined by TMM calculations. Determining the electric field distribution in the SC according to the wavelength is critical, particularly for a light-absorbing device that evolves from opaque to ST characteristics and provides an assessment of how photon harvesting occurs. Indeed, the wavelength at which the reflective top metal contact can reflect the photons and how much of these photons fall into the device’s active region and are harvested provides a relative evaluation of the photovoltaic performance. The distribution of the electric field intensity (|E(z, λ)|^2^) calculated by TMM for the optimally determined ST and opaque OSCs based on PTB7 is given in Fig. 5.

When the distribution of the electric field intensity is analysed for the optimal case offering maximum AVT with the asymmetric MAW (10/5/22 nm) DMD transparent contact system (Fig. 5a), The reflection of the photons travelling in ST-OSC from the top contact back into the active area is minimal since the high AVT requirement is achieved with a relatively thin metal layer. The reflection rate is even lower in the region corresponding to the absorption spectrum characterized by the extinction coefficient of PTB7 and scattered in the VR. Since the metal layer has a higher reflectivity in the NIR region. In addition, the transmission of the thin metal layer causes an electric field intensity distribution in the WO_3_, which has a waveguide effect. This photon transmission to the outer dielectric is crucial. Because WO_3_ has electrochromic properties and its optical properties vary when a voltage is applied^22,26–29^. This has the potential to change many optical properties such as AVT, CRI_ext_ and colour of an optimally structured PTB7-based ST-OSC with a DMD system designed with WO_3_ as the outer dielectric by applying a voltage to the outer dielectric.

The electric field distribution (Fig. 5b) for the PTB7-based ST-OSC offering maximum CRI_ext_ with a thicker metal layer compared to the optimal structure determined for maximum AVT shows that the internal reflection from the top contact is more dominant: The reflection from the top contact improves with increasing thickness of the metal layer to 21 nm. Photons not absorbed in the active region are reflected back to the active region. In particular, the distribution of photons in the 400–700 nm range increases in the active region of PTB7:PC_71_BM. Therefore, this physical process has increased the absorption spectrum given as inset in the Fig. 5. This physical phenomenon harms the AVT by reducing the transmittance. However, high CRI_ext_ can be achieved while simultaneously improving the photovoltaic performance by absorbing more photons in the active region and photon harvesting compared to the optimal ST-OSC offering maximum AVT. The electric field intensity has shown a significant increase in the NIR region, and this increase is highly efficient in the FTO bottom contact and ZnO electron transport layer (ETL) and the parts of the PTB7:PC_71_BM active region close to the ETL. Since FTO, ZnO and PTB7:PC_71_BM are transparent in the NIR region, an increase in the reflectance spectrum of ST-OSC was observed in the NIR region. Therefore, harvesting photons in the NIR region is almost impossible in PTB7-based systems. In the asymmetric MAW (10/5/22 nm) DMD transparent contact system, the reflection from the metal layer, especially in the region where the PTB7:PC_71_BM blend can absorb, substantially increases photon harvesting and photovoltaic performance.

Fullerene-containing PTB7:PC_71_BM OSCs fail to exhibit absorption characteristics for wavelengths greater than 750 nm (Supplementary Fig. 2), and thus no photon harvesting takes place in most of the NIR region. In order to overcome this deficiency, it is important to develop a narrow band gap n-type semiconductors and use them in OSCs. Recently, nearly isostructural non-fullerene electron acceptors have been designed by Lee et al., apart from using solubilizer units with absorbance between 600 and 1100 nm^30^. They designed CTIC-4F, CO1-4F and COTIC-4F molecules with band gaps of 1.3, 1.2 and 1.1 eV, respectively, and obtained strong absorption in NIR^30^. They also designed and synthesized a series of A–π–D–π–A-type NFAs by side-chain engineering to increase NIR absorption in PTB7-based OSCs^31^. Specific molecules include pIO1, o-IO1, p-IO2 and o-IO2 with optical band gaps of 1.34, 1.28, 1.24 and 1.20 eV, respectively^31^. In addition, Vollbrecht et al. obtained EQE up to 1100 nm in a wide spectral range in the OSCs they developed using NFA COTIC-4F and PTB7-based donors^32^. These improvements with the use of NFAs will increase the reabsorption of electromagnetic waves in the NIR, which are reflected back to the active region, especially from the MoO_3_/Ag/WO_3_ transparent top contact and the opaque Ag contact, with the improvement of transmittance in VR and absorption in NIR. Thus, it will be possible to access more efficient and high-transparency ST-OSC designs.

**Figure 6:**
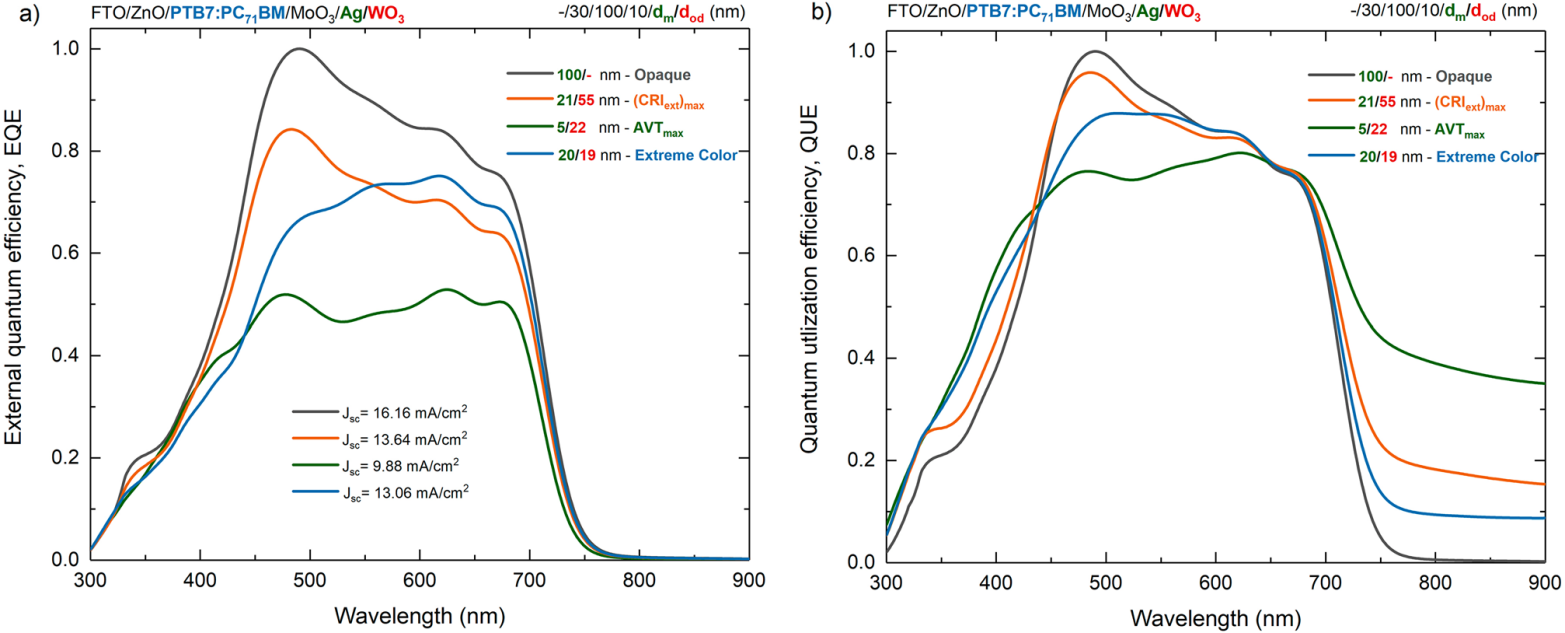
External quantum efficiency and quantum utilization efficiency normalised characteristics of semi-transparent and opaque PTB7-based organic solar cells. (**a**) EQE and (**b**) QUE changes of PTB7-based OSCs with asymmetric MoO_3_/Ag/WO_3_ transparent contact and opaque Ag top contact, optimally determined based on AVT, CRI_ext_ and colour.

The intensity of the electric field distribution in the conventional PTB7-based OSC with thick Ag top contact is greater than the other optimized PTB7-based ST-OSCs. As expected, the high reflectivity of the 100 nm thick Ag top contact is the main reason for this. In fact, at this thickness, no photons with wavelengths other than the low wavelength regime in the VR could be scattered in the metal layer and direct internal reflection occurred. In order to more quantitatively evaluate the effect of ST features on the photovoltaic performance of PTB7-based OSCs, EQE and QUE characteristics should be examined. With EQE, a functional photovoltaic performance for an ST OSC can be determined directly by evaluating the optical reflection and transmission losses in an SC. In addition, non-absorbed and transmitted photons can be effectively utilized in an ST optoelectronic device. For this reason, QUE, which includes the sum of EQE and transmittance, should be evaluated to analyse the optical and electrical properties more effectively in PTB7-based ST-OSC^33^. The EQE and QUE characteristics of the ST and opaque PTB7-based OSCs in which the asymmetric MAW transparent contact system is integrated are presented in Fig. 6a,b, respectively. EQE characteristics were calculated by Eq. (32) based on the electric field distribution within the SC determined by TMM. Therefore, the EQE evaluation is based on optical properties and includes an idealized situation without any transport loss.

**Figure 7:**
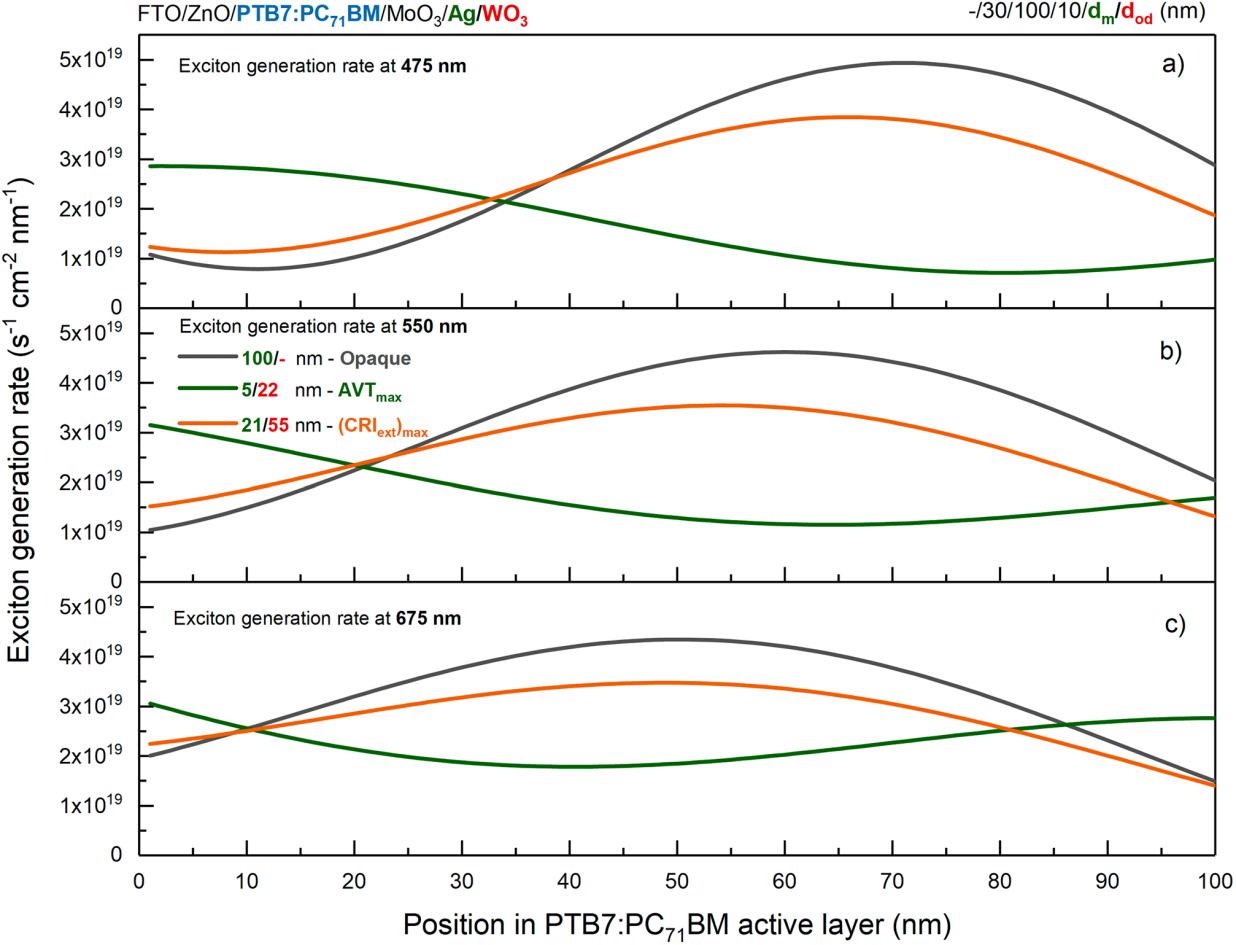
Exciton generation rates of semi-transparent and opaque PTB7-based organic solar cells. Exciton generation rate change is calculated depending on the position in the PTB7:PC_71_BM blend at (**a**) 475 nm, (**b**) 550 nm, and (**c**) 675 nm.

EQE distribution is determined by the AM 1.5G distribution in the short wavelength regime and the absorption characteristics of the PTB7:PC_71_BM blend in the long wavelength regime, and similar distributions have been observed both theoretically and experimentally in the literature^34–37^. As the optimal PTB7-based ST-OSCs evolve from maximum AVT to maximum CRI_ext_ and extreme colour, there is an increase in EQE for all wavelengths due to the improvement in photon harvesting. Mainly this enhancement is maximum for the opaque OSC. The reason is that increasing the thickness of the metal layer in the top transparent contact system MAW DMD system in PTB7-based OSC increases the internal reflection and improves the absorption spectrum of the OSC. In the literature, a similar physical process caused by reflection from the top contact has been observed in PTB7-based OSCs with different contact systems^33,34^. Thus, this effect improved EQE at all wavelengths and increased photovoltaic performance by increasing photon harvesting. In addition, with the increase in the thickness of the metal layer in the distribution of the electric field intensity, the intensification in the VR in the range of 450–550 nm, which corresponds to the PTB7:PC_71_BM active region, increases the EQE in this wavelength range compared to other wavelengths. This may be because the exciton generation rate in the PTB7:PC_71_BM blends for each wavelength is not the same. In addition, in the optimal ST-OSCs with almost the same metal thickness, there is an improvement in the EQE in the 450–550 nm range with the increase in the thickness of the outer dielectric WO_3_, which has a waveguide effect. Because the increase of the dielectric layer from 19 to 55 nm improves the internal reflection in the 450–550 nm range, an enhancement in photovoltaic performance is achieved. The calculated exciton generation rate changes depending on the position in PTB7:PC_71_BM blend at 475 nm, 550 nm and 675 nm for the investigated PTB7-based OSCs are presented in Fig. 7a–c, respectively.

PTB7:PC_71_BM blend, the higher electric field intensity of the electromagnetic waves, especially those at short wavelengths, provides more exciton generation in this region. The improvement in EQE quantitatively shows the effect on the short-circuit current density (J_sc_) values calculated from the EQE distribution with Eq. (32) and hence on the photovoltaic performance of the devices. The J_sc_ values for the optimal ST-OSCs with maximum AVT and CRI_ext_, extreme colour and OSC with opaque characteristics are 9.88 mA/cm^2^, 13.64 mA/cm^2^, 13.06 mA/cm^2^ and 16.15 mA/cm^2^, respectively. The J_sc_ obtained for the opaque PTB7-based OSC is in good agreement with the values obtained for similar structures in the literature, and a decrease in J_sc_ occurs with decreasing thickness of the metal layer^16,33–36^. Additionally, increasing the thickness of the external dielectric at constant metal thickness improves the J_sc_ at 450–550 nm by enhancing the EQE.

We have evaluated QUE, an effective parameter to explain light energy utilization and offer an alternative way for a compelling analysis of the PTB7-based ST-OSCs investigated in the present study (Fig. 6b). QUE not only uniformly describes the device’s conflicting illumination harvesting and transmittance properties but also indirectly reflects the device’s contrasting optical utilization and loss. In the investigated ST and opaque PTB7-based OSCs, the reflection of incident photons at the air/FTO and FTO/ZnO interfaces before reaching the active region, parasitic absorption, non-radiative and intrinsic recombination processes lead to insufficient photon harvesting and the generated free carriers do not contribute to the photocurrent. In particular, the investigation here focuses on how photon harvesting and light energy utilization change with the optical characteristics of the top contact.

The PTB7-based OSC with opaque, thick Ag metal top contact has zero transmittance for all wavelengths. For this reason, the QUE for the opaque OSC is directly equal to the EQE. This property indicates that the opaque PTB7-based OSC utilizes electromagnetic energy only for photon harvesting compared to other optimally presented PTB7-based ST-OSCs. A similar effect has been observed for the transparent to opaque characteristics of PTB7-based devices with similar structures in the literature^38^. In the opaque system, incident photons are either absorbed very efficiently and photon harvesting is increased, or some loss may occur due to optical losses. This physical process is another reason high J_sc_ and photovoltaic performance can be achieved in opaque structures. In the structures determined as optimal for AVT and CRI_ext_, the metal layer is thin and some of the absorbed light energy is sacrificed, making the OSC ST. At the same time, they are reasonably designed and optical losses are also suppressed with optimal design parameters. The reduction of both absorption in the PTB7:PC_71_BM active region and optical losses in the whole structure eventually contributed to the light transmittance, resulting in an increase in QUE over the entire wavelength range (Fig. 6b). For the optimal PTB7-based ST-OSC with maximum CRI_ext_ offering 22.29% AVT, the QUE behaviour is almost identical to the opaque structure up to 700 nm. The main change occurs in the NIR region. Because in this region, the PTB7:PC_71_BM blend has no absorption and no photon harvesting occurs in the device. The optimal PTB7-based ST-OSC offers a maximum AVT of 48.75% and QUE values increase in a wide wavelength regime. For the NIR region, the QUE of the optimal ST-OSC with maximum AVT is considerably higher than the optimal structure with both opaque and high CRI_ext_. In support of the previous evaluation of the metal layer’s contribution to internal reflection, electric field intensity distribution and exciton generation rate, the QUE value at 450–550 nm is lower than the other structures. The optimal PTB7-based ST-OSC with high AVT in the 550–750 nm range offers the same QUE as the other structures indicating that both sufficient photon harvesting and favourable energy utilization with transmittance are achieved.

## Conclusion

The designs of PTB7-based ST-OSCs were carried out with light management engineering approaches and the extraordinary optical limits of ST-OSCs that can be designed with PTB7 were determined. Even in a wide range, such as 1–12 nm of d_m_ and 0–40 nm of d_od_, an AVT greater than 25%, the lower limit for window applications, could be obtained. The maximum AVT is 48.75% for d_m_ = 5 nm and d_od_ = 22 nm. Especially with d_m_, the transmittance in NIR decreased significantly, while a change in NIR and the long wavelength region of VR with d_od_ was observed. The strong electric field observed in metals due to surface charges and the transparency reduced by the plasmonic effect is improved by the thicker outer waveguided WO_3_ dielectric layer. Thus, the AVT is significantly enhanced for a given d_m_ by increasing the d_od_ up to 22 nm.

As a superior performance, ST-OSC could be designed with CRI_ext_ of 90 and above for a wide range of d_m_ and d_od_. The maximum CRI_ext_ value is 99.08 for d_m_ = 21 nm and d_od_ = 55 nm. It was also seen that modifying the current neutral colour perception of a PTB7-based ST-OSC can only be made towards the blue region with the DMD system. As another superior performance, the colour coordinates of the optimal structures offered for AVT and CRI_ext_ are very close to AM 1.5G colour coordinates. Therefore, the ‘colourless’ PTB7-based ST-OSC has been designed for industrial designs that require both high AVT and high CRI_ext_ and have a neutral colour perception. In PTB7-based ST-OSC, colour coordinates that can only be shifted to the blue region can be changed along the Planckian locus up to the 25% AVT limit. The CIE x and y for the most colour-changeable structure (d_m_/d_od_ = 20/19 nm) at the 25% AVT boundary are 0.2410 and 0.2683, respectively. With the asymmetric MAW transparent contact system for the PTB7-based ST-OSC, CCTs could be modified from the 5000 K daylight region to the 12792 K region to sky blue and beyond.

For photovoltaic performance evaluation, photon harvest improved and EQE increased for all wavelengths as optimal PTB7-based ST-OSCs evolved from maximum AVT to structures with maximum CRI_ext_ and extreme colour characteristics. Optimal ST-OSCs with maximum AVT and CRI_ext_, extreme colour and OSC with opaque characteristic J_sc_ values are 9.88 mA/cm^2^, 13.64 mA/cm^2^, 13.06 mA/cm^2^, and 16.15 mA/cm^2^, respectively. Also, for optimal PTB7-based ST-OSC offering maximum AVT, QUE has improved considerably over a wide wavelength regime, especially NIR. Finally, the optimal PTB7-based ST-OSC, which offers maximum AVT, has the same QUE characteristics as the other optimal, showing that both sufficient photon harvesting is achieved and favourable energy use is provided with its transmittance properties.

## Materials and methods

### Materials and solar cell architecture

In the OSC design, transition metal oxides (TMOs) with high transmittance in the visible region, work functions ranging from 2 to 7 eV and wide band gaps were used as carrier blocking layers, i.e. ETL and HTL, respectively. Top transparent contact systems in ST-OSCs are designed as DMDs. The use of TMO as the inner dielectric layer in DMDs presents a highly effective design: TMOs can be used as ETLs and HTLs in the structures in which the DMD system is integrated^6,39^ and thus play an important role in tuning the electrical and optical properties by selecting the suitable dielectric^14–16^.

Examined OSCs are in inverted structure architecture. Thus, illumination is performed from the bottom side (according to Fig. 1). For this reason, fluorine-doped tin oxide (FTO) with high optical transmittance and conductivity and low work function (4.4 eV) was used as a transparent bottom contact material to allow more photons to reach the active region of the OSC from the bottom electrode side^40^. FTO offers superior optical transmittance (> 90%) across the entire VR regardless of fluorine doping^41^. Additionally, FTO can be fabricated at a lower cost than indium-doped tin oxide (ITO), which is often used in conventional structures, and its work function is less dependent on the cleaning procedure^40^. Zinc oxide (ZnO), an n-type metal oxide with high carrier mobility, widely preferred in inverted OSCs, was used as the hole-blocking layer in OSCs. ZnO has a conduction band energy of about − 4.4 eV and a valence band energy of − 7.4 eV. Owing to this band feature, electrons generated in the active region can be transported to ZnO, while holes can be blocked^41^. ZnO is also called the ETL in OSC due to this function. It also offers high optical transmittance in the VR and NIR region due to its wide band gap, allowing photons to reach the active region efficiently^6,7,39^. 30 nm thick ZnO ETL is sufficient for efficient electron transport, hole blocking and optical transmission^42^. In view of these reasons, the thickness of ZnO in the designed OSCs was chosen as 30 nm.

Poly ({4,8-bis[(2-ethylhexyl)oxy]benzo[1,2-b: 4,5-b0]dithiophene-2,6-diyl}{3-fluoro-2-[(2-ethylhexyl)carbonyl]thieno[3,4-b]thiophenediyl}) (PTB7) was used as the donor polymer and (6,6)-phenyl C71 butyric acid methyl ester (PC_71_BM), a fullerene derivative with high electronegativity and superior charge transport properties, was used as the acceptor polymer^43^. BHJ PTB7:PC_71_BM blend is an innovative absorber system with a 0.9 eV band gap, 3.7 eV electron affinity and high carrier mobility. These properties are widely used in ST-OSC structures with advanced optical and photovoltaic performance^4,5,33,34^. The thickness optimization of the BHJ PTB7:PC_71_BM blend, which forms the active region in ST-OSC design, is highly crucial based on parameters such as good absorption characteristics, efficient exciton generation and dissociation, mechanical and environmental stability, and, most of all, good transmittance in VR. These parameters are virtually all thickness dependent and interchangeable with each other^44–46^. Therefore, many studies have been in the literature on optimizing the thickness of the BHJ PTB7:PC_71_BM blend^47,48^. Based on photovoltaic performance parameters such as open circuit voltage (V_oc_), fill factor (FF), PCE, J_sc_ and an essential optical value such as AVT, it has been observed that 100 nm thick BHJ PTB7:PC_71_BM blend shows an optimal performance for ST-OSC^5,42,49^. That is why the absorber layers of ST-OSCs with BHJ PTB7:PC_71_BM blend were designed with a thickness of 100 nm.

Molybdenum trioxide (MoO_3_) was used as HTL and an inner dielectric layer. In opaque OSCs with a thick metal layer, the MoO_3_ layer is only an HTL, whereas, in ST-OSCs, it is both an HTL and the inner dielectric part of the transparent top contact in the DMD structure. In addition, the MoO_3_ HTL layer blocks the penetration of metal into the active region in OSCs. MoO_3_ has high hole mobility and good transparency in the visible region^6,7,14,50^. MoO_3_ has a conduction band energy of about − 2.3 eV and a valence band energy of − 5.3 eV. Due to this band and conductivity properties, holes generated in the active region and separated can be efficiently transported to MoO_3_, whilst electrons can be blocked^41^. So, there are many studies in which MoO_3_ is used as HTL among TCOs with high work function in the design of high-performance optoelectronic devices^14,51,52^.

TMO-based transparent top contact structure for ST-OSCs was designed in asymmetric MoO_3_/Ag/WO_3_ (MAW) DMD configuration (Fig. 1a). Transparent contact designs containing MoO_3_ and WO_3_ are frequently used in screens, tunable mirrors, and energy-saving smart windows. These systems have shown highly promising performances in electrochromic devices, where transparency, colour or other optical properties can be tuned in response to the applied potential^22,26–28^. Therefore, among various TMOs, DMD systems with MoO_3_ and WO_3_ have been extensively investigated in the literature^22,23,53^. In addition, asymmetric MAW transparent contact systems are more functional for inverted ST-OSCs than symmetric MoO_3_/Ag/MoO_3_ and WO_3_/Ag/WO_3_ DMD systems. Because as mentioned above, MoO_3_ as the inner dielectric layer exhibits a suitable HTL performance, whereas WO_3_ used as the outer dielectric has attracted much attention among electrochromic materials due to its chemical stability, strong adhesion to various substrates and high colouring efficiency^14,23,37^. For the electrically optimal state, the thickness of MoO_3_ used as HTL (d_id_:d_MoO3_) significantly affects the photovoltaic performance of the devices, and d_id_ = 10 nm is highly suitable for efficient charge transport^6,7,14,39^. Therefore, d_id_ = 10 nm was chosen for the OSCs investigated in this study.

Ag metal has been used as the top electrode in opaque and ST-OSC structures due to its relatively low absorption coefficient and high electrical conductivity compared to other common metals used in the DMD systems and OSCs^4,5,14,34^. Ag oxidation is prevented by WO_3_ in the asymmetric MAW transparent contact system. Furthermore, the structure’s high refractive index of WO_3_ enabled it to behave as an effective cap layer and waveguided for tunable optical properties^14,23^.

### Calculation of optical spectra

The optical spectra of the ST-OSC and DMD systems designed in this study were calculated using the Transfer matrix method (TMM). TMM is a functional method used in optical and electrical simulations of optoelectronic devices and gives good agreement with experimental results^6,7^. TMM can analyse how the electromagnetic wave propagates inside optoelectronic devices, especially those integrating photonic-based systems such as DMD^6,14,16^ and photonic crystal (PC)^7,15^, where different dielectric media are grown on top of each other.

**Figure 8:**
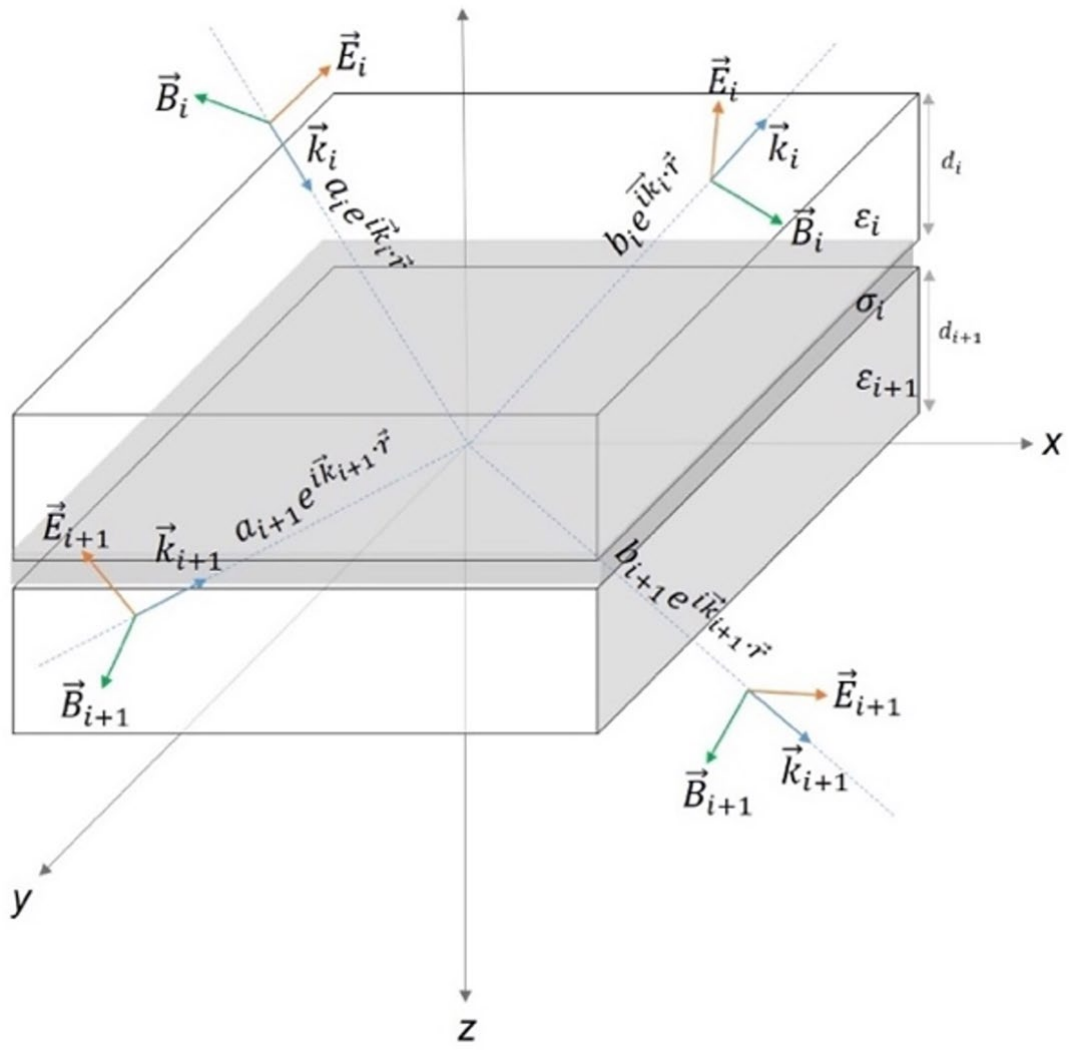
A multilayer dielectric system and a thin metal layer with σ conductivity. According to the order of the dielectric and metal layers, the structure can evolve into ST-OSC, DMD and Photonic crystal systems. Electromagnetic waves reaching and reflecting at the interface are represented by red and blue arrows, respectively.

For a functional optical calculation, three critical properties of materials must be known: absorption coefficients, refractive index spectra and the thickness of each layer (related parameters for all layers are given in Supplementary Fig. 6). Strategically with these parameters, the effective light absorption and the parasitic absorption in each layer can be measured, and then the light intensity passing through each layer can be determined. One of the most established and widely used optical simulation algorithms for both inorganic and organic optoelectronic devices is TMM^4,21^. The spatial evolution of the electric field and magnetic field components in an ST optoelectronic device can be calculated by means of the generated transfer matrix and propagation matrix^7^. The electromagnetic wave’s electric and magnetic field components are connected to each other by the transfer matrix at the interface of the material layers, and the field components propagating in it are connected by the propagation matrix. A multilayer dielectric system and a thin metal layer with σ conductivity is represented in Fig. 8.

Different dielectric systems surround the metal layer and the conductivity (σ) is distributed parallel to the z = 0 planes. Calculating the propagation of the electromagnetic wave along the interfaces formed by conductors, insulators and semiconductors in the whole ST-OSC structure is crucial for determining the optical properties. In order to study the s and p polarisations, it can be assumed that the electromagnetic wave is polarised in the y direction and propagates n the z direction with a particular orientation. The magnetic field components for p polarisation have the following form:

1 $${H}_{1y}={\alpha }_{1}{e}^{i{\overrightarrow{k}}_{1}.\overrightarrow{r}}+{\beta }_{1}{e}^{i{\overrightarrow{k}}_{1}.\overrightarrow{r}}=\left({\alpha }_{1}{e}^{i{k}_{1z}z}+{\beta }_{1}{e}^{-i{k}_{1z}z}\right){e}^{-i{k}_{1x}x}, z<0,$$

2 $${H}_{2y}={\alpha }_{2}{e}^{i{\overrightarrow{k}}_{2}.\overrightarrow{r}}+{\beta }_{2}{e}^{i{\overrightarrow{k}}_{2}.\overrightarrow{r}}=\left({\alpha }_{2}{e}^{i{k}_{2z}z}+{\beta }_{2}{e}^{-i{k}_{2z}z}\right){e}^{-i{k}_{2x}x}, z>0,$$

where ${\overrightarrow{k}}_{i}=\sqrt{{\varepsilon }_{i}}\omega /c (i=1, 2)$ is the wave vector of the electromagnetic wave, ${\varepsilon }_{i} (i=1, 2)$ is the dielectric constant of the medium, $\omega$ is the angular frequency, $c$ is the speed of propagation of the electromagnetic wave in a vacuum and ${\alpha }_{i}$ and ${\beta }_{i} (i=\mathrm{1,2})$ are the coefficients. It is known from Snell’s law that the x components of the wave vectors at the interface in both media will be equal to each other: ${k}_{1x}={k}_{2x}$. Besides, when boundary conditions are applied at the interface for electromagnetic fields^6^:

3 $${\left.{\widehat{n}}_{s}\times \left({\overrightarrow{E}}_{2}-{\overrightarrow{E}}_{1}\right)\right|}_{z=0}=0,$$

4 $${\left.{\widehat{n}}_{s}\times \left({\overrightarrow{H}}_{2}-{\overrightarrow{H}}_{1}\right)\right|}_{z=0}=\overrightarrow{J},$$

equations can be obtained. Where ${\widehat{n}}_{s}$ is the normal unit vector of the surface, $\overrightarrow{J}$ is the surface current density of the metallic layer. By applying the condition $z=0$ and obtaining $\overrightarrow{J}$ by Ohm’s law, the following sets of equations are derived:

5 $$\frac{{k}_{1z}}{{\varepsilon }_{1}}\left({a}_{1}-{b}_{1}\right)-\frac{{k}_{2z}}{{\varepsilon }_{2}}\left({a}_{2}-{b}_{2}\right)=0,$$

6 $$\left({a}_{1}+{b}_{1}\right)-\left({a}_{2}+{b}_{2}\right)={J}_{x},$$

7 $${J}_{x}={\left.\sigma {E}_{x}\right|}_{z=0}=\frac{\sigma {k}_{2z}}{{\varepsilon }_{0}{\varepsilon }_{2}\omega }\left({a}_{2}-{b}_{2}\right),$$

where ${\varepsilon }_{0}$ is the permittivity of the space. With the combination of Eqs. (5), (6) and (7), ${a}_{i}$ and ${b}_{i}$’s ($i=1$) are respectively ${a}_{i+1}$ and ${b}_{i+1}$ and the transition matrix ${M}_{i\to i+1}$ associated by.

8 $$\left(\genfrac{}{}{0pt}{}{{a}_{i}}{{b}_{i}}\right)={M}_{i\to i+1}\left(\genfrac{}{}{0pt}{}{{a}_{i+1}}{{b}_{i+1}}\right).$$

The transition matrix ${M}_{i\to i+1}$, $i=1$ is reduced to the following form for the DMD system with a single metallic layer when $i=1$:

9 $${M}_{1\to 2}=\frac{1}{2}\left(\begin{array}{cc}1+{n}_{p}+{\xi }_{p}& 1-{n}_{p}-{\xi }_{p}\\ 1-{n}_{p}+{\xi }_{p}& 1+{n}_{p}-{\xi }_{p}\end{array}\right),$$

where ${n}_{p}=\frac{{\varepsilon }_{1}{k}_{2z}}{{\varepsilon }_{2}{k}_{1z}}$ and ${\xi }_{p}=\frac{\sigma {k}_{2z}}{{\varepsilon }_{0}{\varepsilon }_{2}\omega }$. For the derivation of the $s$ polarisation, all the derivations followed in the application of Ohm’s law with the magnetic field in the $p$ polarisation and the corresponding boundary conditions can be performed for the electric field. In this way, the transition matrix of the $s$ polarisation becomes as follows:

10 $${M}_{1\to 2}=\frac{1}{2}\left(\begin{array}{cc}1+{n}_{s}+{\xi }_{s}& 1-{n}_{s}+{\xi }_{s}\\ 1-{n}_{s}-{\xi }_{s}& 1+{n}_{s}-{\xi }_{s}\end{array}\right),$$

where ${\mu }_{0}$ is the magnetic permeability of the vacuum and the parameters ${n}_{s}$ and ${\xi }_{s}$ are equal to $\frac{{k}_{2z}}{{k}_{1z}}$ and $\frac{\sigma {\mu }_{0}\omega }{{k}_{1z}}$ respectively. These values depend on the wavelength and incidence angle of the electromagnetic wave. Furthermore, the terms ${n}_{p}$ and ${n}_{s}$ are related to the material’s refractive index and absorption coefficient. The transition matrices for the s and p polarisations are identical except for the difference in sign in the non-diagonal components. A common transition matrix can be constructed by rearranging $j=(s,p)$ and ${\eta }_{p}=1$, ${\eta }_{s}=-1$:

11 $${M}_{1\to 2}=\frac{1}{2}\left(\begin{array}{cc}1+{n}_{j}+{\xi }_{j}& 1-{n}_{j}-{{\eta }_{j}\xi }_{j}\\ 1-{n}_{j}+{{\eta }_{j}\xi }_{j}& 1+{n}_{j}-{\xi }_{j}\end{array}\right).$$

By using the transition matrix at interfaces, the relation between the transmission (t) and reflection coefficients (r) of the electromagnetic wave and the transmittance (T), reflectance (R), and absorbance (A) spectra of the SC structure can be calculated as follows:

12 $${R= \left|r\right|}^{2}={\left|\frac{{M}_{2\to 1}}{{M}_{1\to 1}}\right|}^{2},$$

13 $${T= \left|t\right|}^{2}={\left|\frac{1}{{M}_{1\to 1}}\right|}^{2},$$

14 $$A=1-\left(T+R\right).$$

### Average visible transmittance

The transmittance characteristic of the ST-OSC system can be quantitatively based on the AVT definition. For this aim, a transmittance evaluation is performed based on the photopic response of the human eye. The AVT value is calculated as follows^4,5,54^:

15 $$AVT=\frac{\underset{370\text{ nm}}{\overset{780\text{ nm}}{\int }}T\left(\lambda \right) V\left(\lambda \right) \,{S}_{AM1.5G}\left(\lambda \right) \,d\lambda }{\underset{370\text{ nm}}{\overset{780\text{ nm}}{\int }}V\left(\lambda \right) \,{S}_{AM1.5G}\left(\lambda \right)\, d\lambda },$$

where V(λ) is the photopic response of the human eye, S_AM1.5G_(λ) is the photon flux under AM 1.5G illumination. An AVT value of 25% is considered a lower limit for window applications^54^.

### Colour rendering index (CRI)

The colour rendering index is a measure that takes values in the range of 0–100 and gives the degree to how actual the original colour of objects is under an illuminat^4^. Standard daylight sources or incandescent lamps can achieve a CRI of 100. CRI values of 90 and above are categorized as excellent, and CRI values below 80 are generally classified as poor^7,25^. Test lamp and reference illuminant spectral distributions are used to calculate CRI. This can be done with the spectra of test colour samples (TCS). In this way, the CRI value can be calculated for each test sample, and the CRI of the test lamp can be determined by averaging the CRI values^25^.

ST-OSC and DMD systems are evaluated based on CRI with the tristimulus values obtained from the transmittance spectrum. In this way, the CRI value can be calculated for ST-OSC. For the calculation of colour difference, colorimetric values in uniform colour space are used in CIE 1964 standards^7,14^:

16 $$\begin{array}{*{20}l} {W^{*} = 25Y^{\frac{1}{3}} - 17} \hfill \\ {U^{*} = 13\left( {u - u_{n} } \right)} \hfill \\ {V^{*} = 13\left( {v - v_{n} } \right)} \hfill \\ \end{array} ,$$

where $\mathrm{u}$ and $\mathrm{v}$ are the CIE 1960 colour coordinates. ${u}_{n}$ and ${v}_{n}$ are the CIE 1960 colour coordinates of the illuminator. $\mathrm{u}$ and $\mathrm{v}$ can be determined from tristimulus values or CIE 1960 colour coordinates:

17 $$u=\frac{4X}{X+15Y+3Z}=\frac{4x}{-2x+12y+3},$$

18 $$v=\frac{6Y}{X+15Y+3Z}=\frac{6y}{-2x+12y+3}.$$

The colour coordinates of the ST-OSC system to act as an illuminator in window applications may not match the colour coordinates of the reference illuminator. This discrepancy is corrected by colour matching, where the components of the test source are represented by index $i$ and the components of the reference illuminator by index $k$^25^:

19 $$\begin{array}{*{20}l} {u_{k}^{^{\prime}} = u_{r} } \hfill \\ {v_{k}^{^{\prime}} = v_{r} } \hfill \\ {u_{k,i}^{^{\prime}} = \frac{{10.872 + 0.404c_{r} \frac{{c_{k,i} }}{{c_{k} }} - 4d_{r} \frac{{d_{k,i} }}{{d_{k} }}}}{{16.518 + 1.481c_{r} \frac{{c_{k,i} }}{{c_{k} }} - d_{r} \frac{{d_{k,i} }}{{d_{k} }}}}} \hfill \\ {v_{k,i}^{^{\prime}} = \frac{5.520}{{16.518 + 1.481c_{r} \frac{{c_{k,i} }}{{c_{k} }} - d_{r} \frac{{d_{k,i} }}{{d_{k} }}}}} \hfill \\ \end{array} ,$$

where ${u}_{k}^{^{\prime}}$ and ${v}_{k}^{^{\prime}}$ are the CIE 1960 colour coordinates of the test source after colour adaptation, ${u}_{r}$ and ${v}_{r}$ are the reference illuminant, ${u}_{k,i}^{^{\prime}}$ and ${v}_{k,i}^{^{\prime}}$ are the CIE 1960 colour coordinates of the test colours after colour adaptation. ${c}_{k}$ and ${d}_{k}$ are coefficients obtained from the chromaticity diagram and can be calculated as follows:

20 $$\begin{array}{*{20}l} {c = \frac{4 - u - 10v}{v}} \hfill \\ {d = \frac{1.708v + 0.404 - 1.481u}{v}} \hfill \\ \end{array} .$$

The c and d coefficients can be indexed according to the colour coordinates of both the test source and the reference illuminator. Additionally, the tristimulus values for each test colour and the illuminating source and test source are determined to calculate the W, U and V values in CIE 1964 standards. By modifying the equations with the indices, the chromaticity values for CIE 1964 standards can be presented with the following sets of equations:

21 $$\begin{array}{*{20}l} {W_{r,i}^{*} = 25Y_{r,i}^{1/3} - 17} \hfill \\ {U_{r,i}^{*} = 13W_{r,i}^{*} \left( {u_{r,i} - u_{r} } \right)} \hfill \\ {V_{r,i}^{*} = 13W_{r,i}^{*} \left( {v_{r,i} - v_{r} } \right)} \hfill \\ {W_{k,i}^{*} = 25Y_{k,i}^{1/3} - 17} \hfill \\ {U_{k,i}^{*} = 13W_{k,i}^{*} \left( {u_{k,i} - u_{k} } \right)} \hfill \\ {V_{k,i}^{*} = 13W_{k,i}^{*} \left( {v_{k,i} - v_{k} } \right)} \hfill \\ \end{array} .$$

According to the CIE 1964 uniform colour space, the colour differences of each test colour with respect to the illuminating source and the test source are calculated as follows:

22 $$\Delta {E}_{i}=\sqrt{{({U}_{r,i}^{*}-{U}_{k,i}^{*})}^{2}+{({V}_{r,i}^{*}-{V}_{k,i}^{*})}^{2}+{({W}_{r,i}^{*}-{W}_{k,i}^{*})}^{2}}.$$

The colour rendering indices for all test colours can be calculated with colour differences specific to the test colours:

23 $${R}_{i}=100-4.6\Delta {E}_{i}.$$

The colour rendering index of each test colour can be used to derive an overall colour rendering index.

24 $${R}_{g}=\frac{1}{N}\sum_{i=1}^{N}{R}_{i}.$$

The index $i$ takes different values up to the number of test colours of interest ($N$). The CRI of the test source can be determined on the basis of 8 TCS and 7 additional TCS^7^.

### Colour perception

The colour perception of ST-OSCs is crucial in the definition of optical properties. For this purpose, the International Commission on Illumination (CIE) 1931 (x, y) chromaticity diagram was formed to determine the colour perceived by the human eye from a spectrum^55^. The colour perception of ST-OSCs depends on the added photonic system. Optical manipulations such as the use of the photonic crystal, dielectric mirror (DM) and DMD are widely applied to tune the colour appearance of ST-OSCs^56^.

The neutral colour perception is represented by the “white point” with colour coordinates (0.3333, 0.3333). The standard daylight illuminants D65 and AM 1.5G are given in colour coordinates (0.3128, 0.3290) and (0.3202, 0.3324), respectively, and are used as reference incident light in characterization processes^57^. In the tristimulus system, $X, Y$ and $Z$ values can be calculated by the following equations under VR:

25 $$X=\underset{370\text{ nm}}{\overset{780\text{ nm}}{\int }}\,{S}_{AM1.5G}^{D65}\left(\lambda \right) \,T\left(\lambda \right) \overline{x }\left(\lambda \right) \,d\lambda ,$$

26 $$Y=\underset{370\text{ nm}}{\overset{780\text{ nm}}{\int }}{S}_{AM1.5G}^{D65}\left(\lambda \right)\, T\left(\lambda \right) \overline{y }\left(\lambda \right)\, d\lambda ,$$

27 $$Z=\underset{370\text{ nm}}{\overset{780\text{ nm}}{\int }}{S}_{AM1.5G}^{D65}\left(\lambda \right)\, T\left(\lambda \right) \overline{z }\left(\lambda \right)\, d\lambda .$$

In these equations, ${S}_{AM1.5G}^{D65}$ is the CIE standard D65 illuminant spectrum and the terms $\overline{x }\left(\lambda \right), \overline{y }\left(\lambda \right), \overline{z }\left(\lambda \right)$ are the colour matching functions defined by the CIE protocol (Colour matching function is given in Supplementary Fig. 1). The sum of $X, Y$ and $Z$ is equal to one and the colour coordinates can be simplified to two-dimensional coordinates:

28 $$x=\frac{X}{(X+Y+Z)},$$

29 $$y=\frac{Y}{(X+Y+Z)}.$$

The colour coordinates can be determined by modifying the transmittance spectrum with an optimized ST-OSC design. In such a manner, window applications can be accessed with a transparent or semi-transparent ST-OSC in the targeted colour.

### Correlated colour temperature (CCT)

CCT identifies a specific point along the blackbody curve in the CIE 1931 chromaticity diagram. CCT is used especially to describe white light sources and presents an absolute, one-dimensional measurement system. A “cold colour” with a bluish appearance has a colour temperature above 5000 K, and a “warm colour” with a yellowish appearance has a low colour temperature of about 2700–3000 K^58^. At the same time, CCT for ST optoelectronics is a system that shows their similarity to light emitters in various technological applications^59^.

Small deviations from the blackbody curve occur due to absorption in the atmosphere or the different designs of artificial light sources. That is why most light sources do not present a colour exactly on the blackbody curve. This is also true for natural daylight. Therefore, the colour temperature is described as correlated with a statistical process caused by this deviation. Because of this, it is required to calculate the CCT closest to the blackbody rather than to determine the CCT on the blackbody curve. The amount of deviation is determined by the condition ${\Delta }_{u,v}$ < 0.054 in CIE1960 colour coordinates^7,14^. If ${\Delta }_{u,v}$ > 0.054, i.e. the deviation is high, it can be mathematically associated with a colour temperature value. However, in this case, the light source can no longer be considered “white”. Hence, it is more convenient and functional to present ${\Delta }_{u,v}$ alongside the CCT. The CCT value can be calculated using the MacCamy approximation as follows^60^:

30 $$CCT=-449{ \,m\left(x,y\right)}^{3}+3525\, {m\left(x,y\right)}^{2}+6823.3 \,m(x,y)+5520.33,$$

where $m(x,y)=\left(\frac{x-0.3320}{y-0.1858}\right)$. $x$ and $y$ are CIE1931 color coordinates.

### Generation rate

For each of the wavelengths for which AM 1.5G is responsible, the production of electron–hole pairs can be calculated at any position in the ST-OSC. Most of the production of electron–hole pairs occurs in a region quite close to the surface in the layer where absorption is high. The production rate is a parameter that gives the number of electrons and holes generated due to the absorption of the electromagnetic wave incident on the SC. With the TMM, the optical model used in the study, the electric field intensity (${\left|E\right|}^{2}$) and the photon absorption rate or charge generation rate (G) of the plane electromagnetic waves incident on the SC can be calculated depending on the position and wavelength in the structure as follows^34^:

31 $$G\left(z, \lambda \right)=\frac{2\pi {\varepsilon }_{0}n\left(\lambda \right)k\left(\lambda \right){S}_{AM1.5G}\left(\lambda \right)}{h}{\left|E\left(z, \lambda \right)\right|}^{2},$$

where n(λ) is the refractive index, k(λ) is the extinction coefficient and h is the Planck constant.

### External quantum efficiency and quantum utilization efficiency

EQE is related to the absorption of each photon in the SC and the number of electrons and holes that can be supplied to the external circuit. It is determined by the ratio of the flux of electrons from the SC to the flux of incident photons. EQE can be calculated from the distribution of the electric field intensity in the SC and the optical properties of the materials by the following Eq. (3):

32 $$EQE\left(\lambda \right)=\int \frac{2\pi c{\varepsilon }_{0}n\left(\lambda \right)k\left(\lambda \right)}{\lambda }{\left|E\left(z, \lambda \right)\right|}^{2}dz.$$

In the optimal condition, each photon will produce an electron–hole pair and the free carriers will move towards the depletion region. They will then be separated and collected by the internal electric field. In other words, EQE is usually a photovoltaic performance parameter to describe the use of photons in the SC. The EQE definition was obtained from the spectral and optical properties, as it was made over the distribution of the electric field intensity calculated directly by TMM. Therefore, the calculated EQE provides an idealized assessment that does not include any transport loss. The photons passing through the SC are also beneficial if the SC is ST. Therefore, a new quantum utilization efficiency (QUE), which is the sum of EQE and transmittance, can be defined to analyse the optical and electrical properties of ST-OSCs more effectively^33^:

33 $$QUE\left(\lambda \right)=EQE\left(\lambda \right)+T\left(\lambda \right).$$

The reflection of photons arriving at the ST-OSC at the air/substrate and substrate/ETL interfaces before reaching the active region, parasitic absorption, non-radiative and intrinsic recombination processes lead to insufficient photon harvesting, and the generated carriers do not participate in the current. Therefore, QUE should be less than 90% in the entire spectral range.

### Short-circuit current density

The short-circuit current density (J_sc_) is the current density through the SC when the voltage across the SC is zero; that is, when the solar cell is short-circuited. J_sc_ can be theoretically calculated by the double integral of G over the absorption wavelength range and ST-OSC thickness when the internal quantum efficiency (IQE) value is 100%:

34 $${J}_{sc}=e\iint G\left(z, \lambda \right) \,dz \,d\lambda ,$$

where e is the elementary charge. J_sc_ can also be calculated from EQE by the following expression^34^:

35 $${J}_{sc}=e\int \frac{{\lambda S}_{AM1.5G}\left(\lambda \right)}{hc} EQE\left(\lambda \right)d\lambda ,$$

Since the J_sc_ integrant includes EQE, it represents an idealized situation and provides an evaluation that does not include transport losses.

## Supplementary Information

Supplementary Figures.

## Data Availability

The materials and data that support the findings of this study are available from the corresponding authors on request.
