# Supplementary material for: In-depth analysis on PTB7 based semi-transparent solar cell employing MoO3/Ag/WO3 contact for advanced optical performance and light utilization
Source: Sci Rep. 2023 May 9;13:7548. doi: 10.1038/s41598-023-34507-y (PMC10170103; doi:10.1038/s41598-023-34507-y)
Supplement: Supplementary file 1 — Supplementary Figures. [file 41598_2023_34507_MOESM1_ESM.pdf]

Supplementary Information

In-depth analysis on PTB7 based semi-transparent solar cell employing MoO_3/Ag/WO_3 contact for advanced optical performance and light utilization

Erman Çokduygulular[1*], Çağlar Çetinkaya[2], Serkan Emik[3], Barış Kınacı[4,5]

[1]*Department of Engineering Sciences, Faculty of Engineering, Istanbul University-Cerrahpaşa, TR-34320, Istanbul, Türkiye*

[2]*Physics Department, Faculty of Science, Istanbul University, TR-34134, Istanbul, Türkiye*

[3]*Department of Chemical Engineering, Faculty of Engineering, Istanbul University-Cerrahpaşa, TR-34320, Istanbul, Türkiye*

[4]*Department of Photonics, Faculty of Applied Sciences, Gazi University, TR-06500, Ankara, Türkiye*

[5]*Photonics Application and Research Center, Gazi University, TR-06500, Ankara, Türkiye*

[*]*Correspondence should be addressed to Erman Çokduygulular (email: erman.cokduygulular@iuc.edu.tr)*

Supplementary Figure 1

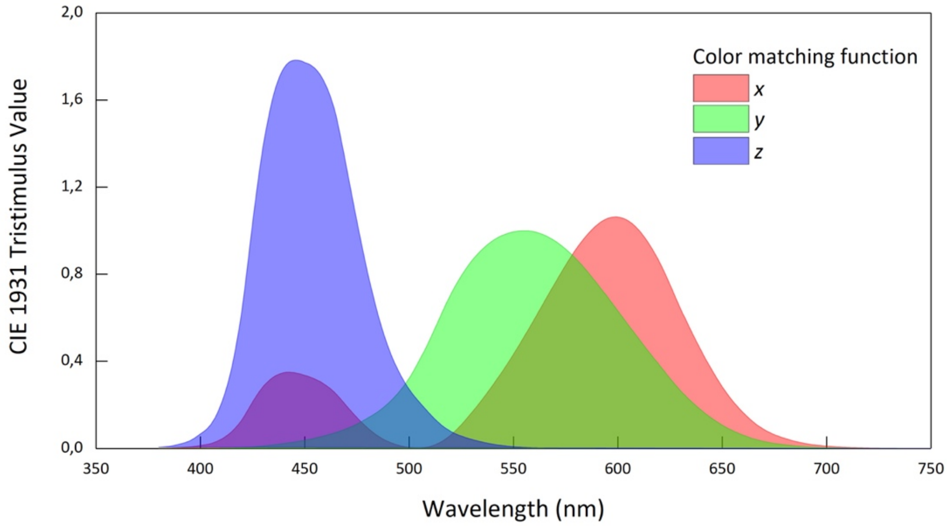

Supplementary Figure 1. Spectral distribution of $\bar{x}(\lambda)$, $\bar{y}(\lambda)$ and $\bar{z}(\lambda)$ colour-matching function.

Supplementary Figure 2

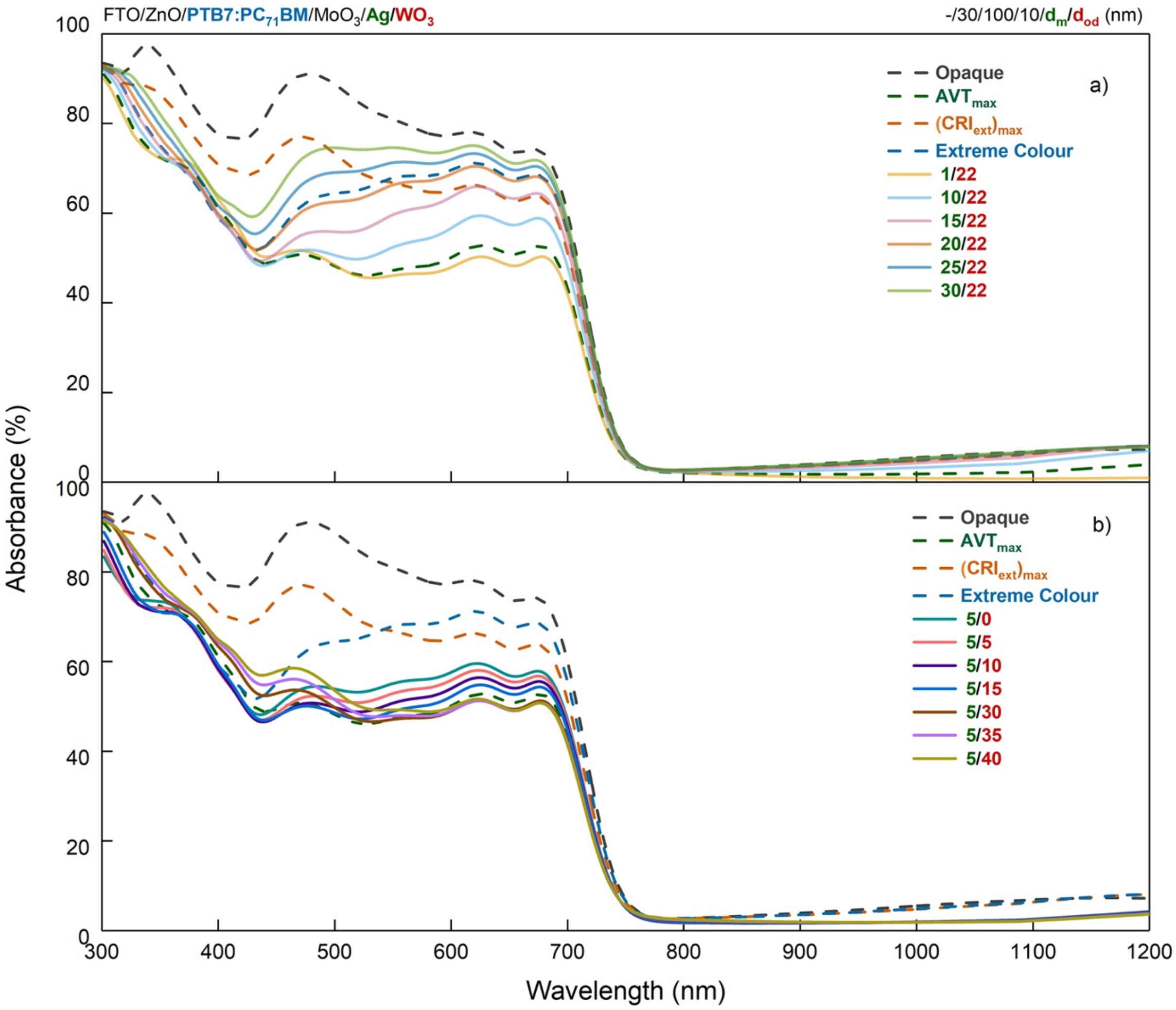

Supplementary Figure 2. Absorption spectra of PTB7-based semi-transparent organic solar cell with asymmetric $MoO_3/Ag/WO_3$ transparent contact system (300-1200 nm). a) according to d_m change and b) according to d_{od} change.

Supplementary Figure 3

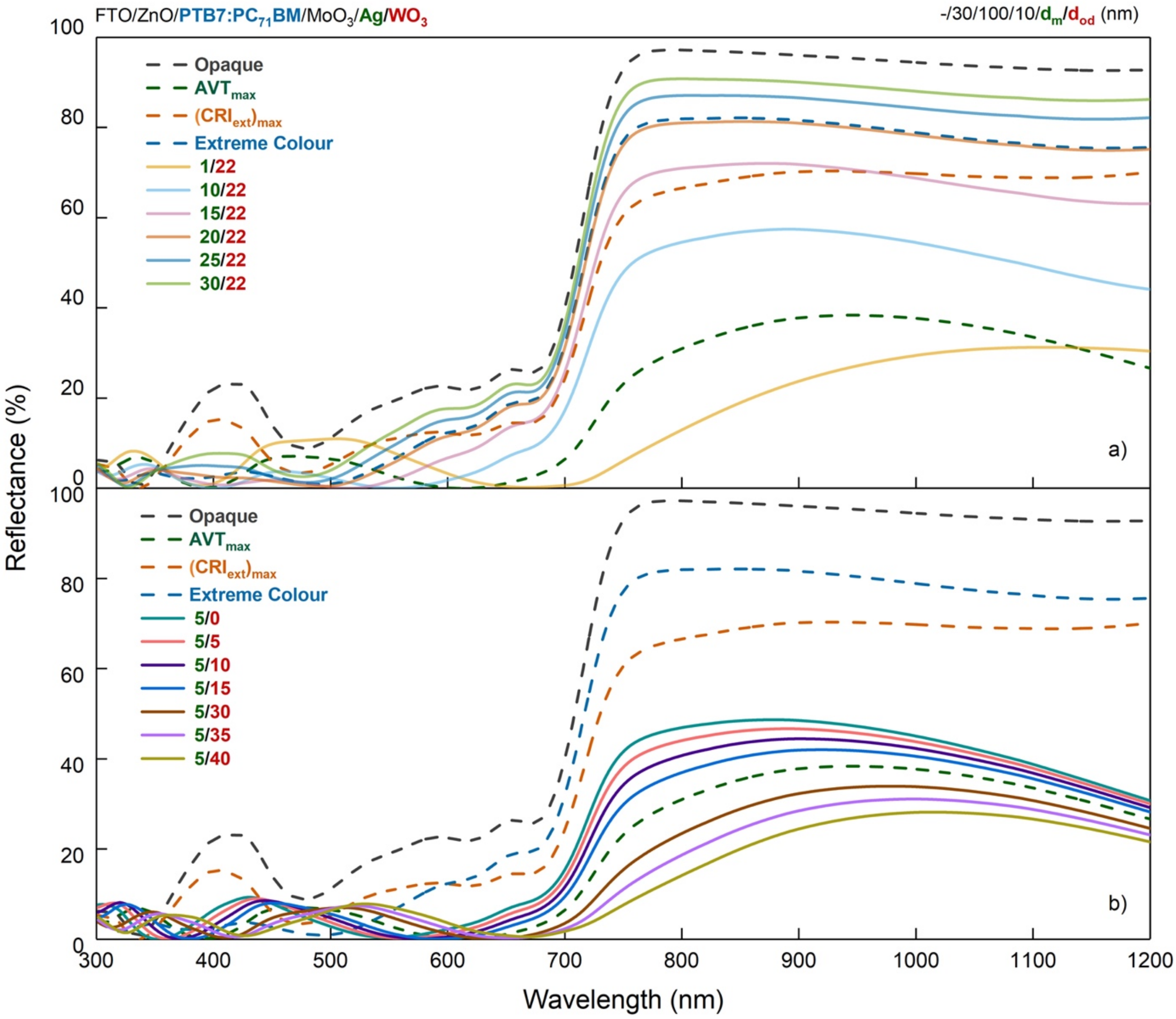

Supplementary Figure 3. Reflectance spectra of PTB7-based semi-transparent organic solar cell with asymmetric MoO_3/Ag/WO_3 transparent contact system (300-1200 nm). a) according to d_m change and b) according to d_{od} change.

Supplementary Figure 4

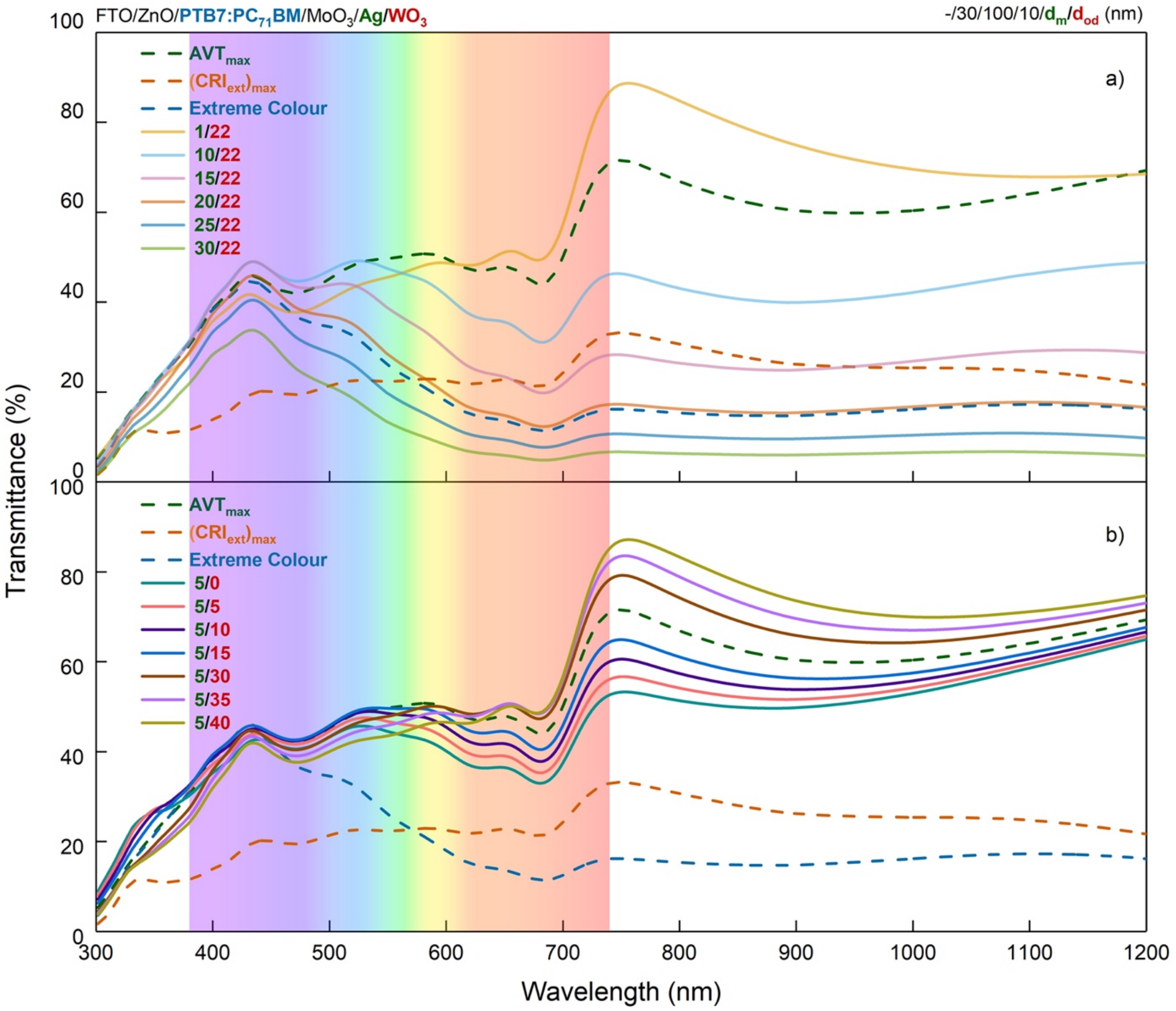

Supplementary Figure 4. Transmittance spectra of PTB7-based semi-transparent organic solar cell with asymmetric MoO_3/Ag/WO_3 transparent contact system (300-1200 nm). a) according to d_m change and b) according to d_{od} change.

Supplementary Figure 5

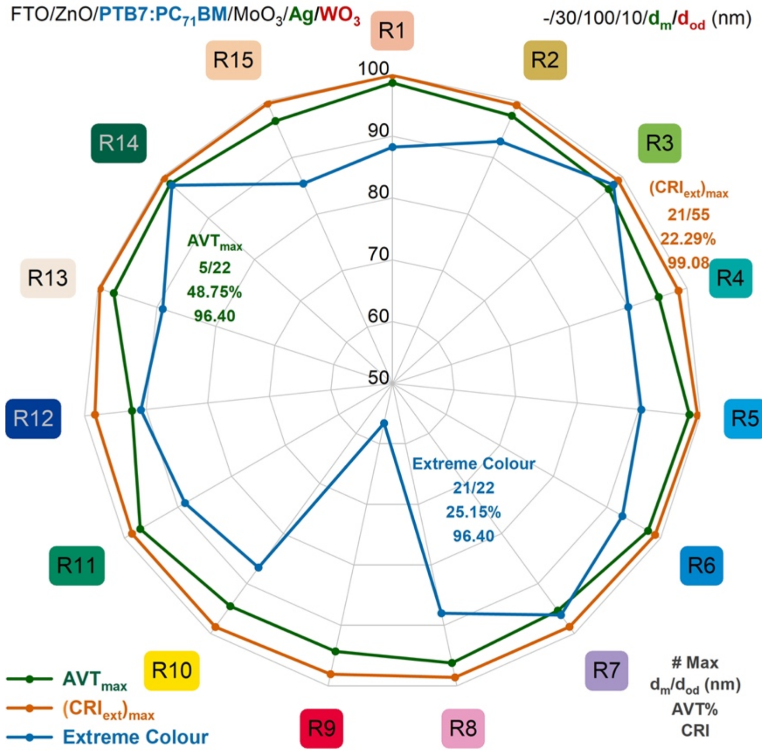

Supplementary Figure 5. Test colour sample values of optimal PTB7-based ST-OSCs offering maximum AVT, CRI_{ext} and extreme colour point. The presented test colour samples are represented in their true colours and are given by R.

Supplementary Figure 6

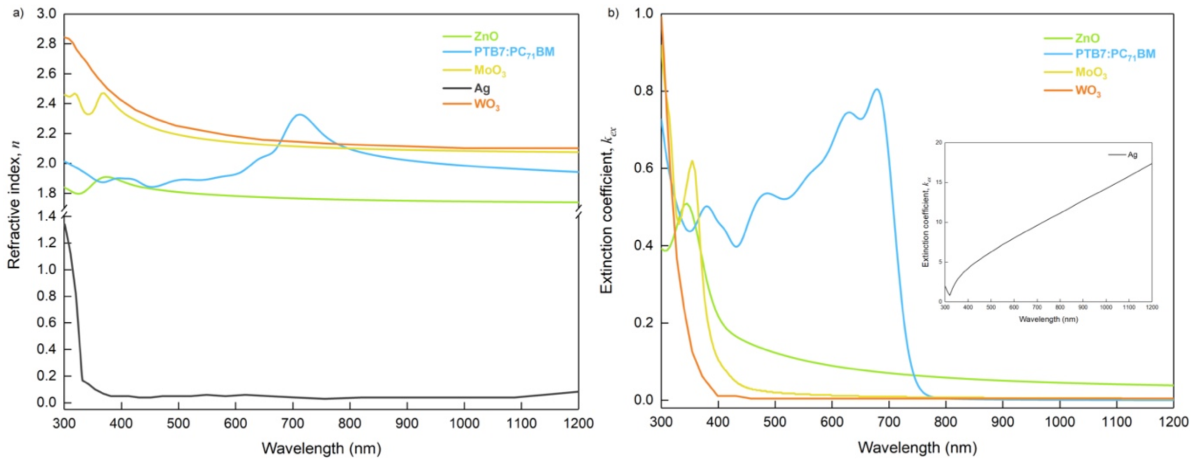

Supplementary Figure 6. Variations of a) refractive indices and b) extinction coefficients of all layers as function of wavelength[1–7].

Supplementary References

1. Çetinkaya, Ç., Çokduygulular, E., Güzelçimen, F. & Kınacı, B. Functional optical design of thickness-optimized transparent conductive dielectric-metal-dielectric plasmonic structure. *Sci Rep* **12**, 8822 (2022).

2. P. B. Johnson and R. W. Christy. Optical Constant of the Nobel Metals. *Phys Rev B* **6**, 4370–4379 (1972).

3. Lajaunie, L., Boucher, F., Dessapt, R. & Moreau, P. Strong anisotropic influence of local-field effects on the dielectric response of α-MoO_3. *Phys Rev B* **88**, 115141 (2013).

4. Kulikova, D. P. *et al.* Optical properties of tungsten trioxide, palladium and platinum thin films for functional nanostructures engineering: erratum. *Opt Express* **28**, 35413 (2020).

5. Aguilar, O., de Castro, S., Godoy, M. P. F. & Rebello Sousa Dias, M. Optoelectronic characterization of $Zn_{1-x}Cd_xO$ thin films as an alternative to photonic crystals in organic solar cells. *Opt Mater Express* **9**, 3638 (2019).

6. Stelling, C. *et al.* Plasmonic nanomeshes: their ambivalent role as transparent electrodes in organic solar cells. *Sci Rep* **7**, 42530 (2017).

7. Çetinkaya, Ç. *et al.* Enhancement of color and photovoltaic performance of semi-transparent organic solar cell via fine-tuned 1D photonic crystal. *Sci Rep* **12**, 1–13 (2022).
